# AI-Guided *De Novo* Design of a Caffeine-Induced Protein Dissociation System

**DOI:** 10.1021/jacs.6c02343

**Published:** 2026-05-25

**Authors:** Tatsuki Nonomura, Brendan McKee, Anna Price, Mingguang Cui, Zaynah Yousuf, Faith Tran, Lian He, Tianlu Wang, Yubin Zhou

**Affiliations:** † Center for Translational Cancer Research, Institute of Biosciences and Technology, Texas A&M University, Houston, Texas 77030, United States; ‡ Department of Translational Medical Sciences, College of Medicine, Texas A&M University, Houston, Texas 77030, United States

## Abstract

Chemically induced proximity (CIP) and chemically disrupted proximity (CDP) technologies have transformed biological research by enabling precise temporal control of protein–protein interactions and cellular functions. However, despite the extensive development of CIP systems, CDP tools with strong translational potential remain comparatively underdeveloped, calling for the need to expand the CDP toolkit. Here, we present a caffeine-operated dissociation system (CODS), a CDP platform activated by caffeine–an inexpensive and widely available small molecule from food or beverages with a well-established safety profile. Using an AI-guided *de novo* protein design framework, we reprogrammed an existing caffeine-responsive CIP module into a ligand-dependent dissociation system. CODS exhibits high sensitivity and rapid functional switching, with an EC_50_ below 90 nM and minute-scale dissociation and reassociation kinetics. We further demonstrate the utility of CODS across diverse cellular contexts, including caffeine-dependent suppression of gene expression, induction of programmed cell death via pyroptosis, and conditional deactivation of chimeric antigen receptor (CAR) T-cell activity. CODS establishes a broadly applicable CDP platform for tunable control of cellular functions and provides a potentially generalizable strategy for engineering chemically controlled dissociation systems from existing CIP architectures.

## Graphical abstract

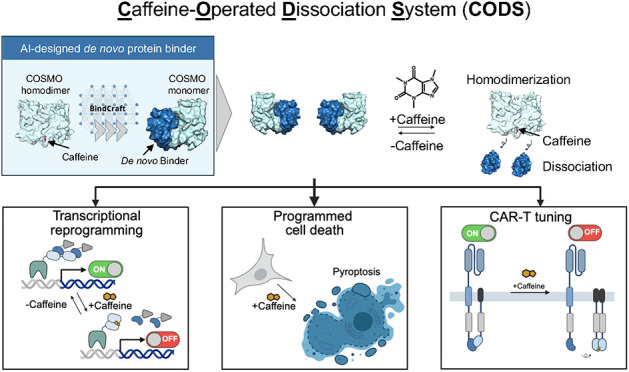

## Introduction

Chemically induced proximity (CIP) systems are foundational tools in synthetic biology, enabling the regulation of biological processes through precise temporal and dose-dependent control of protein–protein interaction (PPI) using small molecules as chemical triggers.^1–4^ In contrast, chemically disrupted proximity (CDP) systems abolish pre-existing PPIs in response to small molecules, providing an orthogonal, on-demand mechanism to terminate biological activity.^2,5^ Despite extensive efforts to expand the repertoire of CIP tools,^2,5–9^ CDP systems remain comparatively underexplored, representing an untapped opportunity for complementary modes of biological regulation. Moreover, for practical clinical translation of CDP technologies, small-molecule inducers with low cost, predictable and favorable safety profiles, and strong preclinical validation are highly desirable. Among currently available CDP inducers,^5,10–13^ several FDA-approved agents, including Venetoclax^10^ and multiple hepatitis C virus NS3/4A protease (NS3a) inhibitors,^5,12^ appear promising. However, Venetoclax targets endogenous BCL-2 family proteins in mammalian cells,^14^ making it unsuitable for applications in normal human cells that depend on these proteins for survival. In addition, NS3a-based CDP systems often show limited reversibility because the relatively large NS3a inhibitors are difficult to wash out from cells.^5,12^ By contrast, caffeine, a widely consumed compound found in beverages such as coffee and tea, exhibits a well-defined pharmacokinetic profile and an established safety record at commonly used doses,^15^ thus making it an attractive alternative to the currently limited set of CDP inducers. Moreover, in our previously developed caffeine-induced homodimerization and heterodimerization systems,^16,17^ caffeine was readily washed out from mammalian cells, suggesting that caffeine-based CDP systems may achieve highly efficient reversibility.

The recent surge in the production and usage of artificial intelligence (AI) models has dramatically accelerated the design of *de novo* protein binders against protein targets,^18–20^ delivering substantial gains in speed and cost efficiency compared with conventional screening-based approaches.^21^ The expansive design space and flexibility enabled by AI-driven protein engineering also makes this approach particularly well suited for constructing synthetic modules for chemogenetic platforms such as chemically induced proximity (CIP)^22–24^ and chemically disrupted proximity (CDP) tools. These advances motivated us to adopt an AI-guided strategy for the development of a caffeine-induced dissociation system.

In the present work, we report the development of a caffeine-operated dissociation system (CODS), which enables rapid caffeine-mediated dissociation of protein complexes through an AI-driven *de novo* protein design pipeline, BindCraft.^18^ After establishing the CODS, we further demonstrated the broad applicability of CODS in controlling diverse biological processes, including transcriptional reprogramming, programmed cell death, and chimeric antigen receptor (CAR) activity modulation in therapeutic immune cells. Overall, this study expands the synthetic biology toolbox with clinically compatible CDP systems and establishes a versatile engineering strategy for the development of CDP modules powered by AI-based protein design.

## Results and Discussion

### Design and Screening of *De Novo* COSMO Binders

In our previous work, we developed a Caffeine-Operated Synthetic Module (COSMO),^16,17^ a system that induces homodimerization of caffeine-binding nanobodies (VHH) upon caffeine addition. Notably, the W104 residue plays a critical role, conferring substantially increased caffeine sensitivity relative to the wild-type VHH.^16^ We hypothesized that designing *de novo* protein binders targeting the caffeine-binding pocket in COSMO would yield a caffeine-induced dissociation system. In this design, caffeine addition induces COSMO homodimerization, which was further expected to competitively disrupt the COSMO-binder interaction and promote dissociation of the designed binders (Figure 1A). To test our hypothesis, we first generated *de novo* miniproteins targeting the caffeine-binding pocket of COSMO employing the BindCraft pipeline^18^ (Figure 1B), which has shown high success rates in converting predicted binder designs to functional *de novo* proteins *in vitro*.^25^ The AlphaFold-predicted structure of COSMO was provided to BindCraft as the design template,^26^ with W104 designated as a hotspot residue. BindCraft yielded 13 miniprotein candidates predicted to engage the caffeine-binding pocket and the homodimerization interface of COSMO (Supporting Table 1 and Figure 1). The 13 miniproteins were ranked based on their corresponding interface-predicted template modeling (ipTM) values, which has been shown to be a crucial metric for identifying positive binders.^26,27^ To identify the true binders responsible for this effect, proteins are typically purified and their binding affinity to COSMO and responsiveness to caffeine are compared. However, this approach is time-consuming, labor-intensive, and costly.

**Figure 1:**
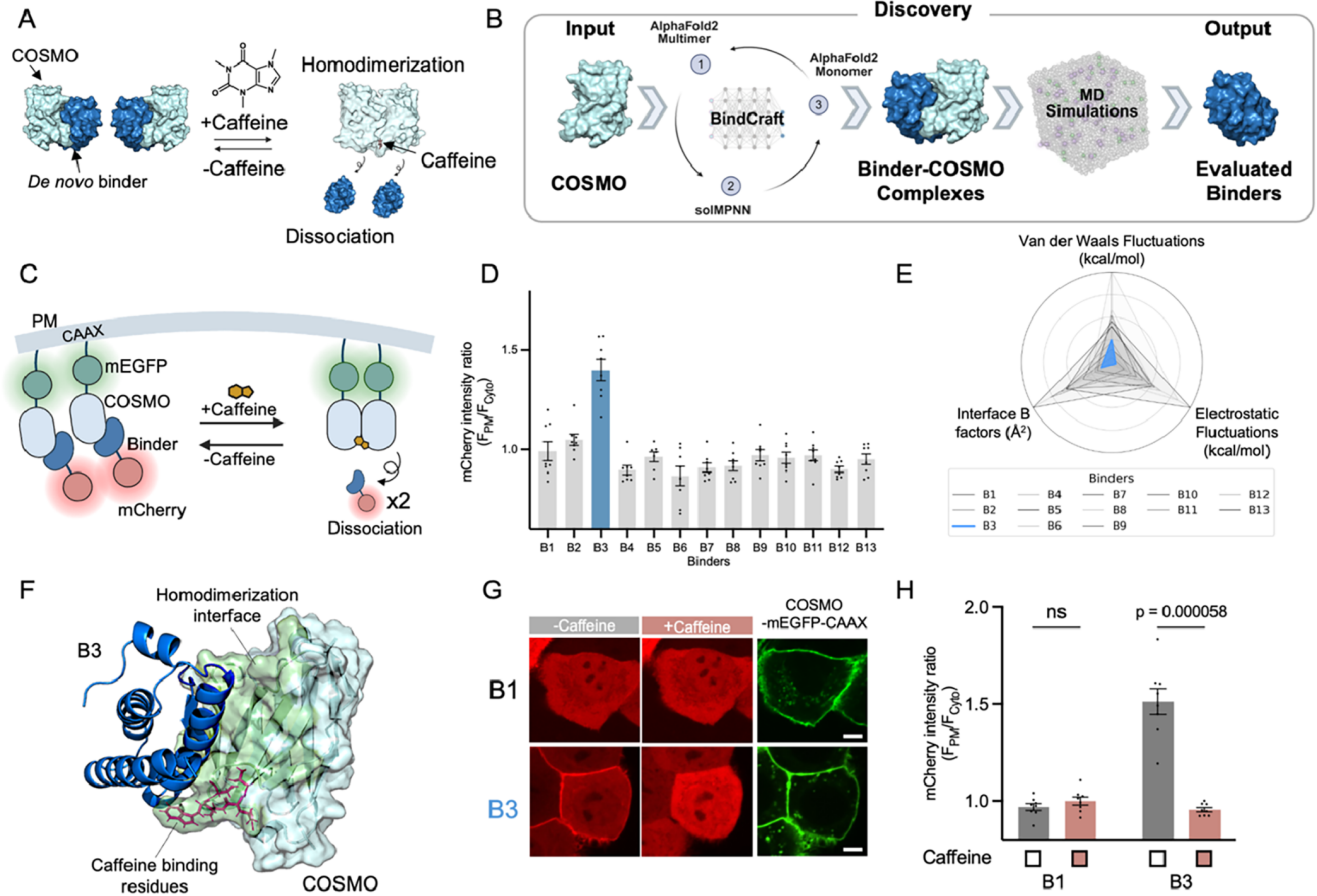
Computational design and experimental validation of COSMO binders. (A) In the caffeine-operated dissociation system (CODS), caffeine triggers COSMO homodimerization, thereby competing for the binder interaction interface and resulting in the dissociation of the *de novo* binders of COSMO. (B) Schematic illustration of the *de novo* binder discovery pipeline utilizing BindCraft for binder design and molecular dynamics (MD) simulations for *in silico* validation. (C) Schematic illustration of the design of the plasma membrane (PM) colocalization assay used to validate the *de novo* binders. (D) Quantification of the plasma membrane-to-cytosol (PM/Cyto) ratio of mCherry fluorescence signal for the 13 predicted binders without caffeine addition. (E) Radar plot of binder interface characteristics. MD simulations were completed for the *de novo* designed binders and assessed using criteria such as van der Waals and electrostatic fluctuations in combination with interface B-factors to provide a holistic evaluation of binding interface stability. Of the binders analyzed, B3 (blue) was identified as the most promising due to low interface conformational and energy fluctuations. For each binder, 4 × 500 ns simulations were completed with an average GPU performance of 202 ns/day. (F) Predicted structure of the COSMO (cyan)-B3 (blue) complex. B3 engages the COSMO homodimerization interface (green surface) and contacts caffeine-binding residues (red sticks). (G) Confocal images of HeLa cells showing colocalization between COSMO-mEGFP-CAAX and the binders before and after treatment with 10 μM caffeine. The non-COSMO binding B1 was used as control. Scale bars, 5 μm. (H) Quantification of caffeine-induced changes in PM colocalization. *n* = 8 cells from three independent biological replicates (mean ± s.e.m.). The *p*-values were calculated using Welch’s *t* test.

To overcome these limitations and better align with future downstream *in vivo* and clinical translation, we developed a cell-based screening strategy using a plasma membrane (PM)-to-cytoplasm colocalization assay, which more faithfully captures protein behavior in a native cellular context and markedly improves screening efficiency (Figure 1C). In this design, COSMO, fused to EGFP, is anchored to the intracellular side of the PM, whereas each candidate binder (B1–B13) is fused to mCherry. Upon interaction between COSMO and the binder, the binder will display clear PM colocalization with COSMO in HeLa cells. By contrast, binders which show no interaction with COSMO will remain diffusely distributed in the cytosol. COSMO-binder interactions were quantified by measuring the degree of colocalization between PM-anchored COSMO and cytosolic mCherry signals in live-cell confocal fluorescence images. Quantitative image analysis identified B3 as the only candidate showing robust PM colocalization with COSMO (Figure 1D), indicating that B3 binds to COSMO in HeLa cells. To investigate the mechanistic basis for the B3 binding performance, we conducted all-atom molecular dynamics (MD) simulations^28,29^ in explicit solvent (Figure 1B). Across four independent replicate trajectories, MD simulations revealed that B3 consistently maintained stable overall contacts with COSMO, showing minimal conformational rearrangements and low fluctuations in both electrostatic and van der Waals interaction energies at interfacing residues in comparison to other binders (Figure 1E). Notably, resulting MD simulations identified B3′s exceptionally stable binding interface as a key distinguishing feature that correlated strongly with its positive *in vitro* performance. For our system, this dynamic stability assessment proved highly predictive of experimental success, where MD simulations served as a valuable validation step for identifying high-quality binders and for *in vitro* validation. Together, these results provide a molecular-level rationale for the enhanced binding stability observed for B3.

Although we successfully identified a positive binder to COSMO, our goal is to develop caffeine-based molecular tools, which requires further determination of whether B3 can dissociate from COSMO upon caffeine treatment. Based on the predicted complex structure of COSMO with B3, the binding interface between B3 and COSMO largely overlaps with the homodimerization interface of COSMO and its caffeine binding residues (Figure 1F). This overlap suggests a strong potential for caffeine to disrupt the COSMO-B3 interaction, thereby releasing B3 from COSMO. As expected, caffeine treatment immediately triggered redistribution of B3 from the PM to the cytosol (Figure 1G,H) in cells coexpressing both binding partners (cytosolic B3 and PM-anchored COSMO). In contrast, the negative control binder, B1, exhibited no appreciable relocalization upon caffeine stimulation. Collectively, these results support our hypothesis that AI and computationally guided screening can identify authentic binders for caffeine-operated dissociation systems, whereby caffeine-induced COSMO homodimerization sterically occludes the B3-binding interface and promotes binder dissociation.

### Optimization of the Leading Binder Guided by *In Silico* Mutational Scanning

B3 displayed detectable colocalization with PM-tethered COSMO; however, a substantial fraction of B3-mCherry remained in the cytosol (Figure 1G), indicating that the B3-COSMO interaction could be further improved. To enhance binding strength, we focused on B3 residues located within 5 Å of COSMO that participate in interface contacts. To improve mutagenesis efficiency and circumvent the labor-intensive process of exhaustively testing all possible variants, we employed Rosetta^30^ to calculate the predicted changes in Gibbs free energy (ΔΔ*G*) for individual point mutations of B3 and to identify substitutions predicted to stabilize the B3-COSMO complex (Figure 2A). The ΔΔ*G* was calculated as the difference in Rosetta binding energy between the mutants and original B3 sequence.^31,32^ Mutations yielding positive ΔΔG values were predicted to destabilize B3 or the B3-COSMO complex, whereas mutations with negative ΔΔ*G* values were predicted to enhance energetic favorability of the interaction. Because Rosetta energies represent relative scores derived from an empirical energy function rather than absolute thermodynamic free energies,^33^ ΔΔ*G* values were used solely to rank mutations by predicted energetic favorability (Figure 2B).

**Figure 2:**
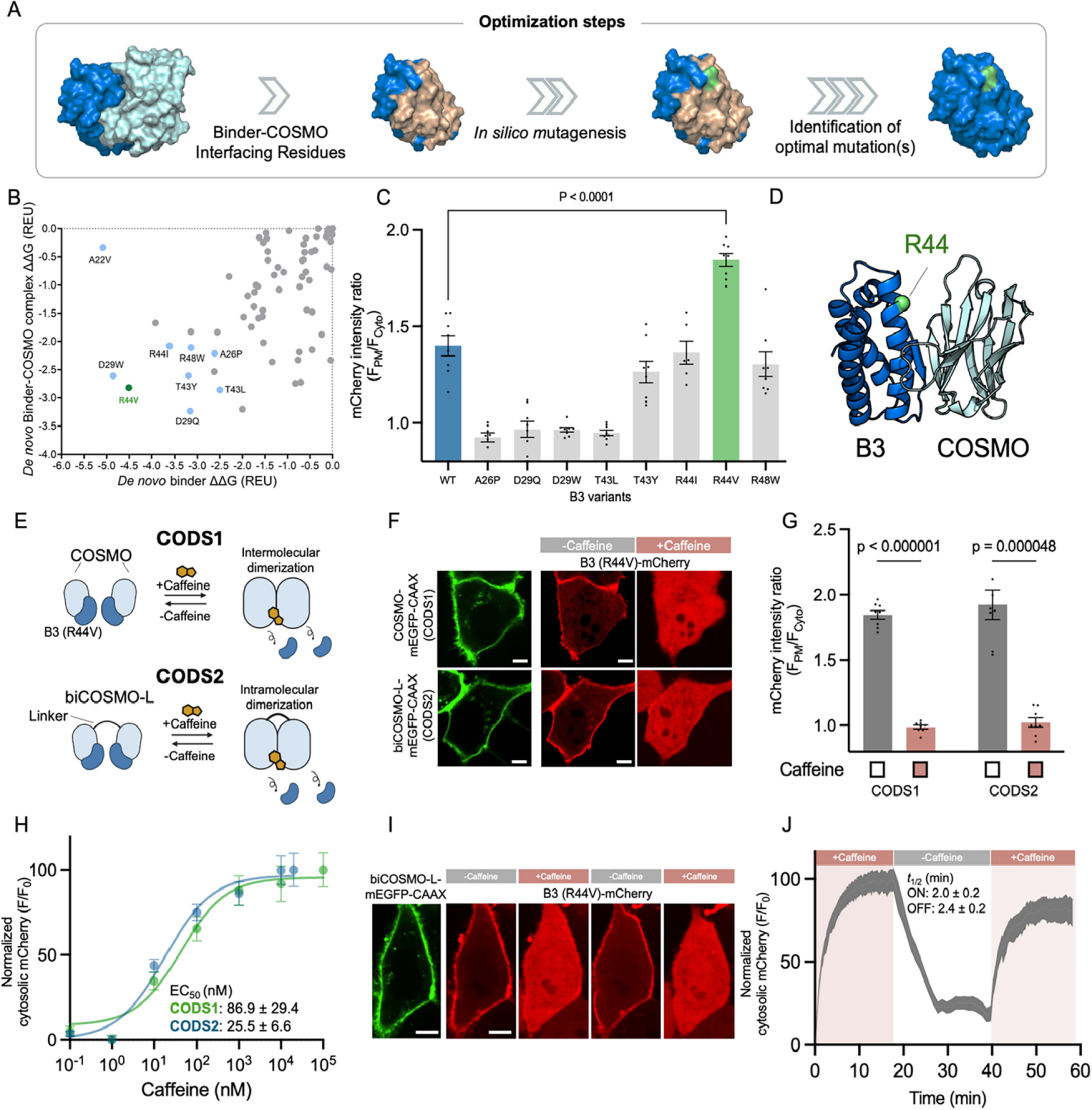
Optimization workflow and performance of computationally optimized CODS. (A) Schematic of the steps used for *de novo* binder optimization via *in silico* mutagenesis. (B) ΔΔ*G* values of B3 point mutations are presented in Rosetta Energy Units (REU), calculated as the resulting energy between the mutant and original sequence. Only mutations that markedly stabilized both the binder and the complex (blue) were considered for *in vitro* validation, and R44V (green) demonstrated the best results. (C) Quantification of the PM/Cyto ratio of mCherry fluorescence signal for the top 8 mutants ranked by computationally predicted ΔΔ*G* values without caffeine treatment. (D) Predicted structures of B3 (blue) and COSMO (gray), highlighting the mutated residue R44 (green). (E) Schematic comparison of CODS1 and CODS2. While COSMO undergoes caffeine-induced intermolecular dimerization and binder dissociation, biCOSMO-L, a bivalent COSMO linked by a long flexible linker, preferentially forms intramolecular dimers. (F) Representative confocal images of HeLa cells coexpressing COSMO-mEGFP-CAAX (top) or biCOSMO-L-mEGFP-CAAX (bottom) with B3­(R44V)-mCherry, illustrating PM colocalization before and after treatment with 10 μM caffeine. (G) Quantification of caffeine-induced changes in PM colocalization between COSMO or biCOSMO-L and B3­(R44V). (H) Dose–response curves of CODS1 and CODS2 expressed in HeLa cells treated with various concentrations of caffeine. Normalized cytosolic mCherry fluorescence intensity was plotted against increasing concentrations of caffeine. (I) Representative confocal images of HeLa cells coexpressing COSMO and B3­(R44V)-mCherry subjected to repeated cycles of 10 μM caffeine treatment and withdrawal. Scale bar, 5 μm. (J) Quantification of changes in cytosolic mCherry signal from B3­(R44V)-mCherry in response to repeated addition and withdrawal of 10 μM caffeine from the culture medium. Data are presented as mean ± s.e.m. (C, G, H, J) For quantified data, *n* = 7 cells per condition from three independent biological replicates. The *p*-values were calculated using Welch’s *t* test.

Mutations predicted to stabilize both the B3 monomer and the B3-COSMO complex were prioritized for cellular validation (Figure 2B). Among the top eight candidates emerging from this dual-filtering strategy, the R44V substitution in B3 exhibited the most favorable predicted energetic improvement, suggesting enhanced intrinsic stability as well as strengthened complex formation. Consistent with these computational predictions, cell-based colocalization assays confirmed that the B3­(R44V) variant displayed enhanced association with COSMO compared with the wild-type B3. This improvement was quantitatively reflected by a significantly increased PM-to-cytosol fluorescence intensity ratio (1.8 for the R44V variant versus 1.4 for wild-type B3; Figure 2C), thereby validating R44V as a functionally stabilizing mutation in a native cellular context (Figure 2D). This computational prescreening approach enabled efficient prioritization of mutation candidates for experimental validation, serving as an *in silico* complement to more comprehensive experimental screening methods such as site saturation mutagenesis.^34^ Next, we evaluated the dissociation behavior of this variant following caffeine treatment. As expected, B3­(R44V)-mCherry rapidly dissociated from PM-tethered COSMO, transitioning from membrane-associated localization to a diffuse cytosolic distribution (Figure 2F,2G).

A distinctive feature of COSMO is its ability to form homodimers upon caffeine stimulation, indicating that this dissociation system inherently involves two COSMO molecules together with an additional interacting partner. This configuration may be advantageous in specific scenarios in which the COSMO-fused target protein dimerizes and becomes functional, whereas the B3­(R44V)-fused target protein dissociates and functions independently upon caffeine treatment. Nevertheless, a more simplified architecture would be desirable, in which only two distinct modules, rather than three, are involved. Our previously developed caffeine-induced intramolecular module, biCOSMO-L, provides an ideal solution to achieve this goal.^16^ By directly replacing COSMO with biCOSMO-L (Figure 2E), we can enforce intramolecular over intermolecular dimerization, as previously validated. Consistent with this design, confocal imaging revealed that mCherry-B3­(R44V) colocalized with PM-anchored biCOSMO-L in the absence of caffeine, whereas caffeine addition abolished their interaction and induced redistribution of mCherry-B3­(R44V) into the cytosol (Figure 2F,G, and Supporting Video 1). Based on these properties, we designated the optimized variants as CODS1 (COSMO + B3­(R44V)) and CODS2 (biCOSMO-L + B3­(R44V)), respectively.

### Characterization of CODS

Given the robust performance of both CODS variants as caffeine-induced dissociation tools, we next characterized their pharmacological and kinetic properties by quantifying EC_50_ values, reversibility, and response kinetics (Figure 2H–J). Consistent with COSMO behavior, CODS1 exhibited an EC_50_ of 86.9 ± 29.4 nM, comparable to that of the original caffeine-induced homodimerization system COSMO (EC_50_ = 95.1 ± 1.2 nM).^16^ In contrast, CODS2 displayed a lower EC_50_ of 25.5 ± 6.6 nM, reflecting the higher efficiency of intramolecular interaction mediated by biCOSMO-L relative to the intermolecular dimerization required for COSMO (Figure 2H). Most importantly, this operating range of CODS1 and 2 falls within clinically safe levels and remains well below the concentrations associated with caffeine addiction or intoxication in the mM range, which can cause severe insomnia or dizziness.^35^ Furthermore, these systems demonstrated robust reversibility over repeated cycles of dissociation and reassociation (Figure 2I,J), with half-lives of 2.0 ± 0.2 min for dissociation and 2.4 ± 0.2 min for reassociation. Notably, these kinetics were slightly slower than those observed for COSMO (0.5–1.5 min),^16^ likely due to the additional energetic barrier associated with displacing the binder during caffeine-induced COSMO homodimerization.

To compare the performance of CODS with existing CDP tools, we conducted our established plasma membrane-to-cytosol colocalization assay for representative CDP systems, including NS3a/ANR^5^, and CDH-3 ^11^, whose corresponding ligands are clinically approved or under clinical evaluation (Supporting Figures 2 and 3). NS3a­(H1) and its binder LD6 exhibited clear plasma membrane localization prior to ligand addition, and comparable *F*_PM_/*F*_cyto_ ratio to CODS. However, they displayed relatively slower dissociation kinetics (Supporting Figures 2 and 3). Moreover, reassociation following ligand washout was not evident for both systems, even under more stringent ligand washout conditions than those used for CODS. Together, these observations suggest that CODS enables faster response dynamics and improved reversibility relative to the current representative CDP systems evaluated here.

Next, we evaluated the compatibility of CODS with our previously established chemically induced dimerization (CID) system, the salicylic acid (SA)-mediated binary association system (SAMBA).^9^ HeLa cells coexpressing CODS1 and SAMBA components were sequentially treated with caffeine and salicylic acid (Supporting Figure 4A–C). Upon caffeine addition, B3­(R44V) was released from the plasma membrane, consistent with disruption of CODS-mediated protein–protein interactions. Subsequent treatment with salicylic acid induced recruitment of the SAMBA­(N) component to the plasma membrane. Notably, owing to the highly reversible nature of both CODS and SAMBA, these effects were fully reversed upon removal of both ligands from the medium. Collectively, these results demonstrate that CODS can operate alongside existing CID systems without functional interference, supporting its potential for combinatorial control of cellular processes.

### Caffeine-Mediated Termination of Gene Expression

With the CODS in hand, we first tested whether this system could be used to control gene expression, where target gene expression could be regulated by the addition and removal of caffeine. To this end, we modified the classical tetracycline-controlled transcriptional activator (tTA) system^36^ by incorporating CODS2 to confer caffeine sensitivity. Specifically, TetR was fused to biCOSMO-L, and the VP16 transcriptional coactivator was fused to B3­(R44V). In this design, TetR-biCOSMO-L binds the TetR response element (TRE), and CODS2-mediated association with VP16-B3­(R44V) drives transcription of the downstream target gene. Upon caffeine treatment, disruption of CODS2 is expected to release VP16 from the promoter-bound complex, thereby terminating transcription of target genes (Figure 3A). Consistent with this mechanism, caffeine administration in transfected cells expressing this CODS2-tTA system reduced luciferase expression by 20.5-fold (Figure 3B).

**Figure 3:**
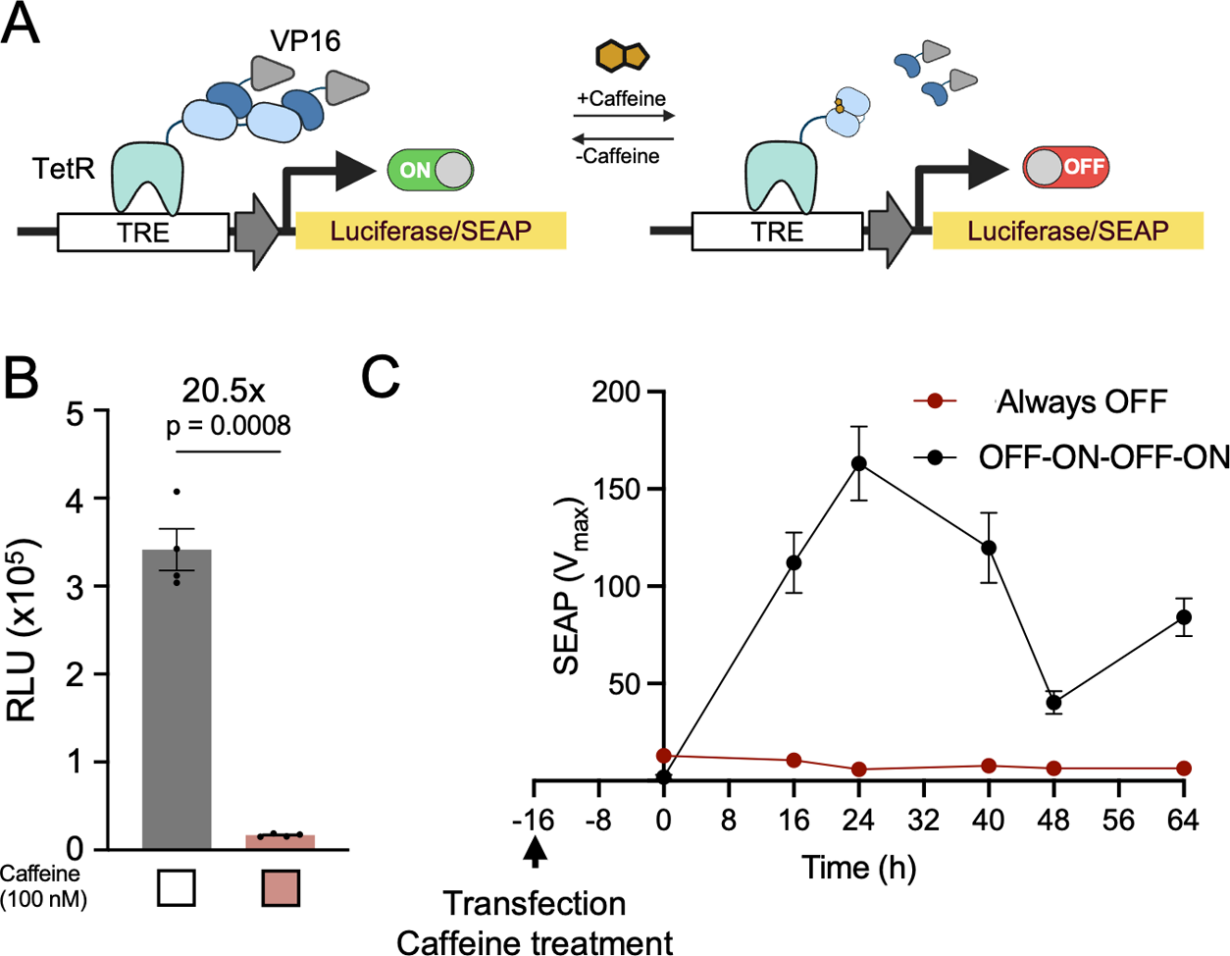
Caffeine-mediated reversal of gene expression. (A) Schematic illustration of a transcriptional reprogramming system using caffeine as a suppressor. Transcription is ON in the absence of caffeine, resulting in constitutive activation of reporter genes (luciferase or a secreted alkaline phosphatase SEAP). The addition of caffeine disrupts the CODS2 interaction, terminating gene transcription. (B) Quantification of CODS2-tTA induced luciferase expression in HEK293T cells with or without caffeine addition (100 nM). (C) Quantification of SEAP secretion from HEK293T cells under constant or cycled caffeine treatment. Following transfection, cells were treated with 10 μM caffeine. Culture media were collected and replaced with fresh media at the indicated time points for SEAP measurement. For the cycled treatment condition, cells were maintained in caffeine-free media during 0–16 h and 40–64 h, and in caffeine-containing media during 16–40 h. Data are presented as mean ± s.e.m. (*n* = 4 independent biological replicates).

To assess reversibility, cells were subjected to sequential caffeine addition and washout, and the secretion of secreted alkaline phosphatase (SEAP) was measured to evaluate caffeine-dependent regulation of gene expression, independent of pre-existing protein levels. Caffeine addition rapidly suppressed transcriptional output, whereas caffeine withdrawal restored SEAP secretion (Figure 3C). This response was reproducible over multiple treatment cycles, further confirming the reversibility of caffeine-induced tuning of exogenous gene expression relying on CODS2.

### Caffeine-Induced Pyroptosis Using CODS

Having successfully implemented CODS as an OFF-switch to enable caffeine-inducible shutdown of gene expression, we next asked whether this system could be repurposed as an ON-switch for biomedical applications. To this end, we employed CODS to control pyroptosis, a highly inflammatory form of programmed cell death.^37–40^ Gasdermin D (GSDMD), the terminal effector of pyroptosis, is composed of an N-terminal pore-forming domain (GSDMDnt) and a C-terminal autoinhibitory domain (GSDMDct) linked by a protease-cleavable linker.^41–44^ Under basal conditions, full-length GSDMD is inactive in the cytosol. Upon activation of upstream inflammatory caspases, cleavage of the linker releases GSDMDnt from autoinhibition,^45^ allowing it to oligomerize and form membrane pores, ultimately leading to cell swelling and plasma membrane rupture (Figure 4A).

**Figure 4:**
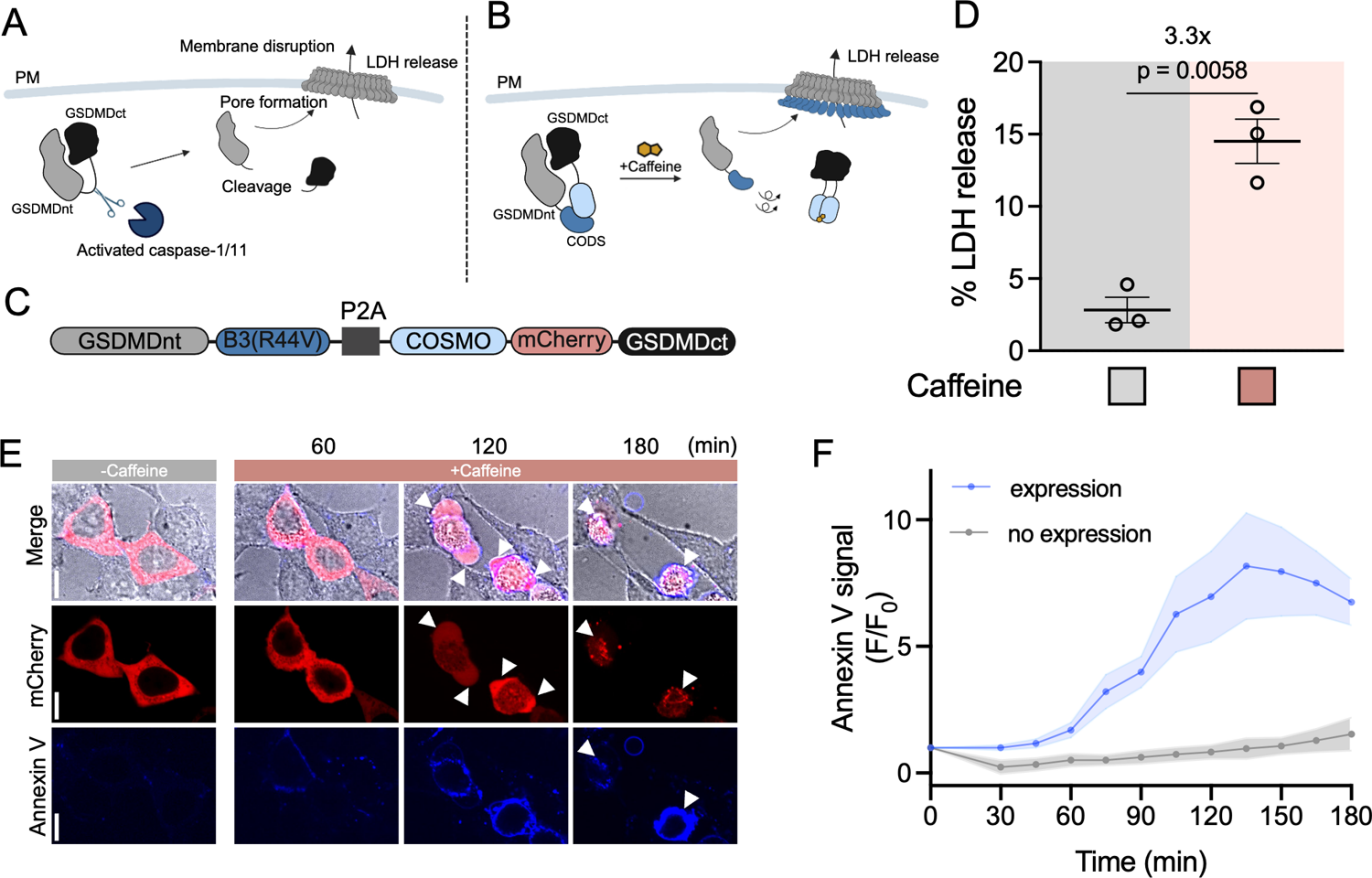
Caffeine-mediated conditional control of pyroptosis using CODS. (A) Schematic illustration of gasdermin D (GSDMD) activation and pore formation during pyroptosis. (B) Schematic illustration of caffeine-induced pyroptosis. In the absence of caffeine, the N-terminal domain of gasdermin D (GSDMDnt) is held in proximity to its C-terminal domain (GSDMDct) via the CODS1 interaction. Addition of caffeine disrupts the CODS1 interaction, releasing GSDMDnt to form membrane pores and ultimately trigger pyroptosis. (C) The CODS1-GSDMD construct used in this study. (D) Quantification of lactate dehydrogenase (LDH) release from HEK293T cells expressing the construct at the indicated time points following caffeine treatment (10 μM). (E) Confocal images of HEK293T cells expressing the construct at the indicated time points following caffeine treatment (10 μM). Cells were stained with Annexin V to visualize pyroptotic cells. White arrowheads indicate pyroptotic bubble formation. Scale bar,10 μm. (F) Quantification of Annexin V staining on the plasma membrane of cells with (light blue) or without (gray) expression of the construct at the indicated time points. Data are presented as mean ± s.e.m.; *n* = 3 (for D) or 8 (for F) independent biological replicates. The *p*-value was calculated using Welch’s *t* test.

To rewire GSDMD activation into a caffeine-responsive trigger independent of linker cleavage, we engineered a CODS1-based system. Specifically, GSDMDnt was fused to B3­(R44V), whereas GSDMDct was fused to COSMO. Both fusion proteins were encoded in a P2A bicistronic expression vector to ensure equimolar coexpression of the two components (Figure 4B,C). In the absence of caffeine, CODS1-mediated association between B3­(R44V) and COSMO enforces an autoinhibited state by functionally tethering GSDMDnt to GSDMDct. Upon caffeine addition, disruption of CODS1 releases GSDMDnt, thereby enabling pore formation and subsequent induction of pyroptotic cell death (Figure 4B).

To quantify cytotoxicity, we measured lactate dehydrogenase (LDH) release into the culture medium as a readout of PM damage. Cells expressing the CODS1-GSDMD construct exhibited a marked increase in LDH release upon caffeine treatment (Figure 4D). Consistent with these findings, live-cell confocal imaging revealed a rapid loss of membrane integrity upon caffeine addition. Cell death initiation was detectable as early as approximately 60 min, with near-complete disruption of intact cell membrane by approximately 3 h, as indicated by annexin V labeling. As stringent control, cells lacking CODS1-GSDMD expression showed no appreciable changes in cell morphology or annexin V signals (Figure 4E,F). In addition, using mEGFP-tagged GSDMDnt-B3­(R44V), we observed clustering of GSDMDnt at the plasma membrane after caffeine treatment, supporting its functional activation (Supporting Figure 5). Collectively, these results demonstrate that CODS1 can be harnessed as a caffeine-controlled ON-switch for pyroptosis, thereby expanding its utility for precise and orthogonal modulation of inflammatory programmed cell death pathways.

### CODS as a Safety OFF-Switch for CAR T-Cell Activity

CDP-based CAR T cell design strategies have emerged as a clinically motivated approach to introduce pharmacological control over CAR signaling, enabling transient suppression of CAR T cell activity to mitigate treatment-associated toxicities such as cytokine release syndrome (CRS) and neurotoxicity.^46–48^ Prior studies have demonstrated that incorporating chemically inducible “breaks” can provide CAR T-cells with periods of functional rest, thereby improving safety without permanently compromising therapeutic potential.^10,49^

Among candidate small-molecule regulators, caffeine represents a particularly attractive choice for clinical translation due to its extensive history of human use, well-characterized safety profile, favorable pharmacokinetics, easy accessibility, and low cost.^15^ Motivated by these considerations, we sought to integrate CODS into a chimeric antigen receptor (CAR) to establish a caffeine-dependent safety OFF switch for CAR T cell activity.

Compared with widely explored small-molecule ON-switch strategies that require ligand-dependent activation of CAR signaling,^9,50–53^ a dissociation-based OFF-switch architecture offers several clinically relevant advantages. CAR signaling remains active by default in the absence of ligand, eliminating the need for continuous drug administration to maintain therapeutic efficacy. Importantly, rapid suppression of CAR signaling can be achieved upon ligand administration, whereas ON-switch systems typically rely on ligand withdrawal and intracellular clearance, leading to delayed deactivation. Collectively, these features position the CODS-based OFF-switch CAR as a practical and clinically translatable strategy for improving the safety and controllability of CAR T-cell therapies.

To establish proof of concept, we generated split CAR constructs by fusing CODS components to two complementary fragments of an anti-CD19 CAR (Figure 5A,B). In the absence of caffeine, CODS-mediated association reconstitutes the split CAR complex, enabling tumor antigen-dependent T-cell activation. Upon caffeine treatment, CODS dissociation disrupts CAR assembly and attenuates downstream signaling, thereby turning off engineered T cell activity (Figure 5A). To optimize this design, we constructed a panel of split CAR configurations (Figure 5B). Part 1 comprised an anti-CD19 single-chain variable fragment (scFv), a transmembrane (TM) domain, a 4–1BB costimulatory domain, and an EYFP-tagged biCOSMO-L or B3­(R44V), designed to maintain the CAR module as a monomer in the active state. Part 2 contained the DAP10 (a transmembrane domain of adaptor protein), mCherry, a 4–1BB costimulatory domain, the T-cell receptor CD3ζ signaling subunit, and either the B3­(R44V) or COSMO module.

**Figure 5:**
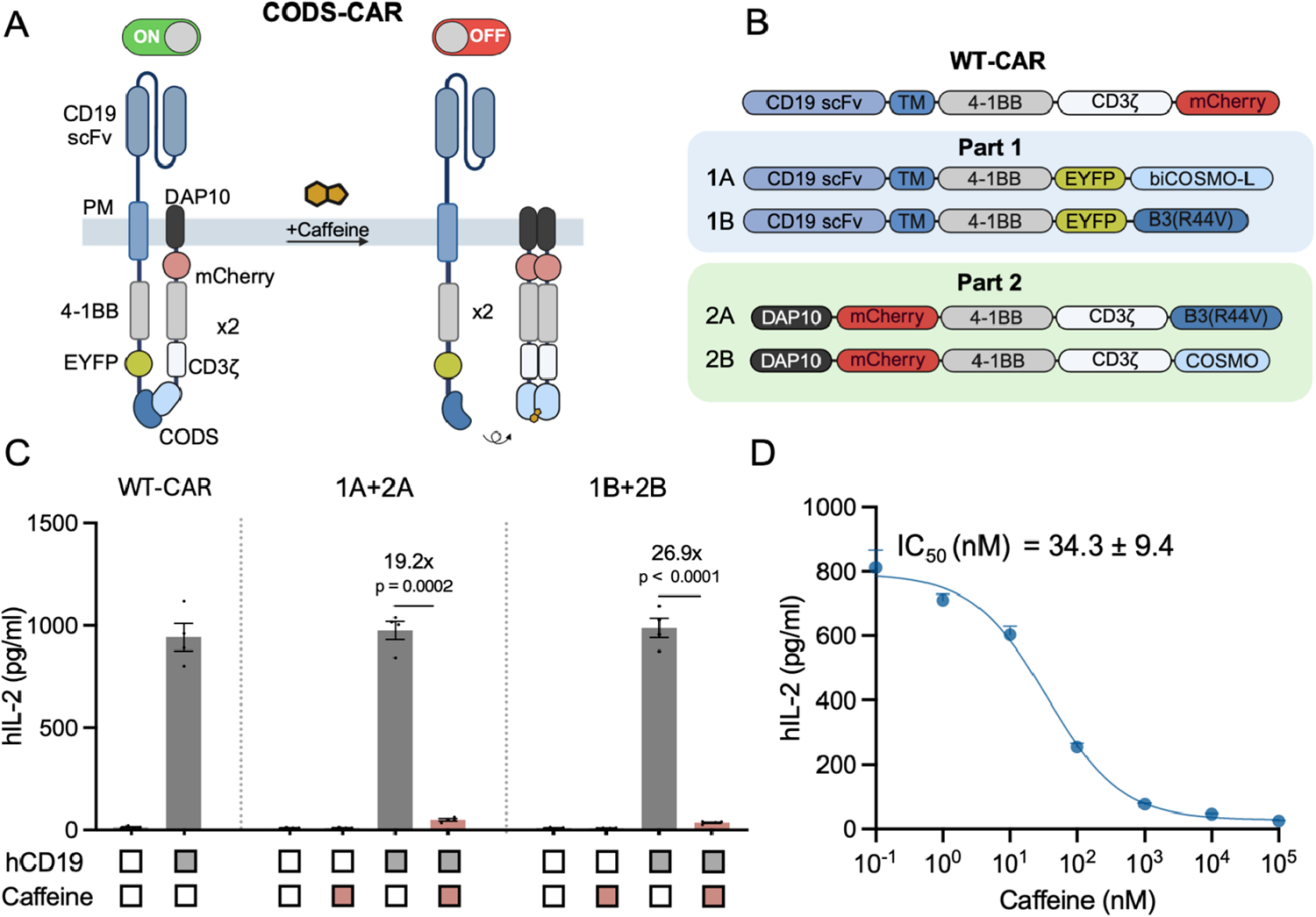
Design of CODS-CAR T cells for caffeine-induced deactivation of T-cell activity. (A) Schematic illustration of the mechanism of caffeine-mediated inactivation of CODS-CAR T cells (constructs 1B and 2B). In the absence of caffeine, the split CAR system is reconstituted and functional via the CODS interaction. Upon caffeine treatment, COSMO dimerizes, and the binder dissociates, leading to CAR splitting and loss of activation signaling. (B) CAR constructs used in this study. (C) Quantification of IL-2 production in Jurkat T-cells stably expressing the indicated constructs. Cells were cocultured with K562 (hCD19^–^) or Raji (hCD19^+^) cells with or without caffeine treatment (10 μM). (D) Dose-dependent IL-2 production in CODS-CAR T cells cocultured with Raji cells at the indicated caffeine concentrations (IC_50_ = 34.3 ± 9.4 nM). Data are presented as mean ± s.e.m.; *n* = 4 independent biological replicates. The *p*-values were calculated using Welch’s *t* test.

To identify the most effective Part 1/Part 2 configuration for caffeine-induced suppression of CAR T-cell activity, we lentivirally transduced Jurkat T-cells with each construct combination. CAR function was evaluated by measuring interleukin-2 (IL-2) secretion, a canonical marker of T-cell activation, following coculture with either human CD19 (hCD19), negative K562 leukemia cells or hCD19^+^ Raji lymphoma cells (Figure 5C). Among the tested configurations, the 1A+2A pair produced IL-2 levels comparable to wild-type (WT) CAR in the absence of caffeine but retained appreciable residual activity upon caffeine treatment. In contrast, the 1B+2B configuration maintained robust IL-2 secretion under caffeine-free conditions yet exhibited near-complete suppression of IL-2 production following caffeine addition, corresponding to a 26.9-fold reduction relative to the untreated state, substantially greater than that observed with the 1A+2A combination (19.2-fold).

We attribute this difference to the underlying assembly stoichiometry of the two designs. In the 1A+2A configuration, a single 1A unit is predicted to simultaneously engage two 2A units, requiring caffeine-induced dissociation of both interactions to fully suppress CAR signaling. By contrast, the 1B+2B design follows a one-to-one interaction model, allowing more efficient and complete disruption of CAR assembly upon caffeine treatment. Based on its favorable combination of strong antigen-dependent activation and efficient, caffeine-triggered shutdown, we selected the 1B+2B configuration as the lead CODS-CAR design for subsequent experiments.

CODS-CAR inactivation exhibited clear caffeine dose dependence, with an IC_50_ of 34.3 ± 9.4 nM (Figure 5D). As mentioned earlier, oral intake of a standard cup of coffee has been reported to yield peak plasma caffeine concentrations of ∼10 μM within ∼1.2 h, and levels around ∼2 μM can be maintained even 12 h after ingestion.^54^ These concentrations are several orders of magnitude higher than the IC_50_ of CODS-CAR, indicating that effective suppression can be achieved at physiologically attainable caffeine levels and sustained for at least 12 h. Based on these pharmacokinetic profiles, it is reasonable to anticipate that approximately once-daily caffeine intake would be sufficient to induce and maintain CAR activity in the OFF state for CODS-CAR-based therapies under most conditions, even without accounting for caffeine metabolism. This operating range is among the most sensitive reported for caffeine-responsive CAR T control systems.^55,56^ Taken together, CODS offers a potential safety switch for engineered cell therapies, supporting its potential application in malignant cancer treatment to improve therapeutic control and safety.

## Conclusion

In summary, we report the first caffeine-operated dissociation system (CODS), which enables rapid chemical disruption of protein complexes through an AI-guided *de novo* binder design pipeline combined with *in silico* optimization. Conceptually, CODS differs fundamentally from existing AI-enabled ligand-responsive systems. Rather than relying on direct ligand-mediated disruption of protein–protein interactions, CODS exploits ligand-induced homodimerization to competitively occlude binding interfaces, thereby displacing prebound interaction partners. This indirect, competition-based mechanism substantially enhances dissociation efficiency and expands the accessible design space for chemically disrupted proximity (CDP) systems. Through affinity optimization of the *de novo* binder, CODS achieves a balance between stable complex formation under basal conditions and efficient ligand-induced dissociation, resulting in minimal leakiness in the absence of caffeine and complete disruption upon ligand addition. Functionally, CODS supports inducible transcriptional regulation, conditional pyroptotic membrane permeabilization, and caffeine-dependent tuning of CAR T-cells with physiologically relevant caffeine concentrations. Furthermore, the EC_50_ of CODS (<0.1 μM caffeine in cells) lies well below plasma concentrations achievable *in vivo*. In humans, oral caffeine intake (such as a cup of coffee or a can of Cola) typically produces peak plasma levels of ∼10 μM with a half-life of several hours,^54,57^ indicating that CODS can be robustly activated within clinically relevant and safe dosing ranges. Although caffeine pharmacokinetics in rodents are characterized by faster clearance and lower peak plasma levels,^58^ micromolar concentrations remain readily attainable, supporting both effective activation of CODS and sufficient persistence of its functional effects in preclinical animal models. Leveraging caffeine’s well-established safety profile, favorable pharmacokinetics, and global accessibility, CODS represents a clinically compatible, small-molecule-controlled CDP platform. More broadly, this work establishes AI-enabled protein design as a general and modular strategy for engineering pharmacologically gated dissociation systems with broad applications in synthetic and chemical biology, as well as in the development of next-generation cell-based therapeutics.

## Supplementary Material





## Notes

Detailed materials and methods; amino acid sequences of the predicted binders and COSMO; predicted structures of COSMO-Binder complexes; plasma membrane-to-cytosol translocation assay for the CDH-3 dissociation system; caffeine-induced clustering of GSDMDnt-B3­(R44V)-mEGFP (PDF)
Caffeine-induced dissociation of B3­(R44V) from plasma membrane-anchored biCOSMO-L (Video 1) (MP4)

## Abbreviations

CIP: chemically induced proximity
CDP: chemically disrupted proximity
CODS: caffeine-operated dissociation system
CAR: chimeric antigen receptor
COSMO: caffeine-operated synthetic module
PM: plasma membrane
SEAP: secreted alkaline phosphatase
GSDMD: gasdermin D
ipTM: interface-predicted template modeling
CRS: cytokine release syndrome.
