# Supplementary material for: AI-Guided De Novo Design of a Caffeine-Induced Protein Dissociation System
Source: J Am Chem Soc. 2026 May 25;148(27):28266–75. doi: 10.1021/jacs.6c02343 (PMC13294833; doi:10.1021/jacs.6c02343)
Supplement: Supplementary file 1 [file ja6c02343_si_001.pdf]

Supporting information for

"AI-guided *de novo* design of a caffeine-induced protein dissociation system"

Tatsuki Nonomura[1, †], Brendan McKee[1†], Anna Price[1], Mingguang Cui[1], Zaynah Yousuf[1], Faith Tran[1], Lian He[1], Tianlu Wang[1,*], and Yubin Zhou[1,2,3*]

[1]Center for Translational Cancer Research, Institute of Biosciences and Technology, Texas A&M University, Houston, TX, 77030, USA

[2]Department of Medical Physiology, College of Medicine, Texas A&M University, Bryan, TX 77807, USA

[3]Department of Translational Medical Sciences, College of Medicine, Texas A&M University, Houston, TX, 77030, USA

Contents:

- Materials and Methods
- Supplementary Table 1
- Supplementary Figures 1-5
- Supplementary Note
- Caption for Supplementary Video
- References

Materials and Methods

Materials and reagents

Caffeine was obtained from Sigma-Aldrich, and 10 mM stock solutions in DMSO were prepared prior to use. Molecular cloning and plasmid construction were performed using KOD Hot Start DNA polymerase (EMD Millipore), along with restriction enzymes, a T4 DNA ligase kit (New England Biolabs), and NEBuilder HiFi DNA Assembly (New England Biolabs). Oligonucleotides were synthesized by Sigma-Aldrich. Synthetic DNA fragments were purchased from Genewiz.

HeLa and HEK293T cell lines were sourced from ATCC and maintained in DMEM (Thermo Fisher Scientific) supplemented with 10% fetal bovine serum (Cytiva) and 1% penicillin-streptomycin (Corning). An NFAT-Luc reporter Jurkat cell line (jktl-nfat) was purchased from Invitrogen. Human cancer cell lines (K562 myelogenous leukemia cells (CCL-243) and Raji cell lymphoblasts (CCL-86)) were purchased from ATCC and cultured in RPMI 1640 medium with l-glutamine supplemented (Corning) with 10% fetal bovine serum (Omega Scientific), and 1% penicillin-streptomycin (Corning). Polybrene was purchased from (EMD Millipore).

Four-well 35-mm glass-bottom dishes, clear flat-bottom 96-well microplates, and 24-well cell culture plates were purchased from Cellvis. Transfections were carried out using Lipofectamine 3000 (Thermo Fisher Scientific) or iMFectin DNA transfection reagent (GeneDEPOT). Bright-Glo Luciferase Assay System (Promega) was used for luciferase reporter assays. SEAP measurements employed Diethanolamine Substrate Buffer (5×) and p-nitrophenyl phosphate (PNPP) tablets, both from Thermo Fisher Scientific. Annexin V (BioLegend) was used for cell death imaging. Pyroptotic cytotoxicity was quantified using an LDH assay kit (MedChemExpress). ELISA was performed using Human IL-2 ELISA kit (Biolegend).

Plasmid construction

Plasmid construction was implemented using standard restriction enzyme digestion and ligation techniques, HiFi assembly or PCR extension methods.

Binder-mCherry constructs were generated by inserting the synthesized cDNA encoding binders into the mCherry-N1 vector using NheI and XhoI restriction enzymes. COSMO-mEGFP-CAAX and biCOSMO-L-mEGFP-CAAX were constructed by inserting COSMO or biCOSMO-L and CAAX sequences[1] into mEGFP-N1 vector using the NEBuilder HiFi DNA Assembly method.

Plasmids for mutagenesis screening were generated by using the QuikChange Site Directed Mutagenesis Kit. To construct plasmids for CODS2-tTA gene expression, mCherry-TetR, biCOSMO-L, and B3(R44V) and VP16 sequences are inserted into pTriex vector using NEBuilder HiFi DNA Assembly method to yield mCherry-TetR-biCOSMO-L and B3(R44V)-VP16, respectively. Then nuclear localization sequence (NLS) was added to each

plasmid using PCR extension methods. To construct GSDMD construct, B3(R44V) and COSMO sequence was replaced with LOV2 and ZDK sequence, respectively, from LiPOP2 construct.[2] The constructs for CODS-CAR were generated based on the prior split CAR system[3] by inserting CODS into pWPXL vector backbone using restriction enzymes AscI and BstBI. All plasmids were verified by Sanger sequencing (Quintara).

Cell culture and transfection

For all cell lines, cells were conditioned at 37 °C with 5% CO_2. For fluorescence imaging experiments, cells were seeded in 35-mm glass-bottom dishes (Cellvis). When cells reach about 50-70% confluency on the next day, transfection was performed using the Lipofectamine 3000 reagent, following the manufacturer's instructions. Then, 6 h post-transfection, cells were replenished with the DMEM with 10% FBS and 1% penicillin-streptomycin cocktail. At 16-24 h post-transfection, cells were transferred to the microscope stage for imaging.

Live-cell imaging and image analysis

Fluorescence imaging was performed on a Nikon Ti2 Inverted microscope equipped with a Yokogawa W-1 dual spinning disk scan head, Micro-Scanner for photo-stimulation and stage top incubator, along with a live-cell culture cage to maintain the temperature at 37 °C with 5% CO_2. Confocal images were acquired using a Nikon 60x 1.40 NA oil-immersion objective.

To screen COSMO binders and B3 mutants, HeLa cells pre-seeded on 35-mm glass-bottom dishes were co-transfected with COSMO-mEGFP-CAAX (300 ng) and binder-mCherry (200 ng) plasmids. Cells were imaged 16–24 h post-transfection. For caffeine-induced dissociation assays, caffeine (prepared in DMEM) was added to the dish during imaging to a final concentration of 10 μM.

The PM-to-cytosol fluorescence ratio (F_{PM}/F_{Cyto}) was quantified using the Intensity Line Profile function in Nikon Elements software. Regions of interest (ROIs) were manually selected along the plasma membrane and in the cytosol for individual cells, and the corresponding fluorescence intensities were extracted. Data were analyzed and plotted using Prism (v10, GraphPad).

The EC_{50} values of CODS1 and CODS2 were determined by incubating HeLa cells in DMEM (10% FBS) containing various concentrations of caffeine until the fluorescence response reached saturation. Changes in cytosolic mCherry fluorescence intensity were quantified as F/F_0 using the Intensity Line Profile function in Nikon Elements software, where F_0 denotes baseline fluorescence prior to caffeine addition. Dose-response curves were

fitted using a four-parameter logistic model (agonist vs response-variable slope) in the Prism software (v10, GraphPad). For each titration curve, eight cells were analyzed, and all experiments were independently repeated three times.

To assess reversibility of CODS2, cells were subjected to repeated cycles of caffeine addition and washout while monitoring cytosolic mCherry fluorescence. After saturation following caffeine addition (10 μM), the medium was removed, and cells were washed three times with caffeine-free DMEM. After fluorescence returned to baseline, DMEM containing caffeine was reintroduced, and cycles were repeated as indicated. Data was analyzed using a custom MATLAB code in which cell masks were created using Cellpose 3.1[4, 5] and the 'cyto2' model[5] to track cytosolic mCherry intensity. Resulting data was plotted using Prism (v10, GraphPad).

Luciferase reporter gene assay

HEK293T cells were seeded in 24-well plates (Greiner Bio-One) and transfected with NLS-mCherry-TetR-biCOSMO-L (300 ng), NLS-B3(R44V)-VP16 (100 ng), and the TRE-luciferase reporter plasmid (50 ng). For the caffeine-treated group, caffeine (100 nM) was added to the culture medium immediately after transfection. After incubation for 16 h, cells were resuspended in PBS, and 100 μL of the suspension was transferred to a clear-bottom 96-well plate. Subsequently, 100 μL of Bright-Glo Luciferase Assay reagent (Promega) was added, and luminescence was measured using a Cytation 5 microplate reader (BioTek). Data were analyzed and plotted using Prism (v10, GraphPad).

SEAP reporter gene assay

24-well plates were pre-coated with 0.01% poly-L-lysine prior to seeding HEK293T cells. Cells were transfected with NLS-mCherry-TetR-biCOSMO-L (300 ng), NLS-B3(R44V)-VP16 (100 ng), and TRE-SEAP reporter plasmid (50 ng). Immediately after transfection, cells were treated with caffeine (10 μM) for 16 h, followed by washout with PBS and re-addition of caffeine during a 64 h culture period. Supernatants were collected at the indicated time points and clarified by centrifugation at 15,800 × g for 5 min. Fresh medium with or without caffeine was replenished after collection. Clarified supernatants were incubated at 65 °C for 1 h to inactivate endogenous alkaline phosphatases. Heat-inactivated samples (40 μL) were incubated with L-homoarginine at 37 °C for 10 min, followed by addition of 60 μL of SEAP substrate to initiate the reaction. Absorbance at 405 nm was recorded every 60 s for 1 h using a Cytation 5 plate reader. Data acquisition and kinetic analysis were performed using Gen5 software (BioTek) and plotted using Prism (v10, GraphPad).

Lentivirus packaging and transduction

Lenti-X cells (ATCC) were co-transfected with lentiviral packaging and envelope plasmids (psPAX2 and pMD2.G) and a CAR/split CAR-encoding lentiviral transfer vector (pWPXL) using iMFectin DNA transfection reagent (GeneDEPOT). Virus-containing supernatants were harvested at 48 and 72 h post-transfection and filtered through 0.45 μm syringe filters to remove cell debris. Lenti-X Concentrator (Takara) was added to the filtered supernatant at a 1:3 (v/v) ratio and incubated overnight at 4 °C, followed by centrifugation at 1,500 × g for 1 h. The viral pellet was resuspended in DMEM and stored at -80 °C until use. For transduction of Jurkat T cells, virus and 4-6 μg /ml polybrene were added to 6-well plates, then centrifuged at 2,000g for 2 h at 32 °C, then cells were incubated at 37 °C with 5% CO_2 prior to downstream assays.

ELISA measurements of cytokine production

Jurkat T cells expressing either conventional WT anti-CD19 CAR or CODS-based split CAR constructs (1×10^5 cells per well) were co-cultured with cognate $CD19^+$ Raji cells or noncognate $CD19^-$ K562 cells at an effector-to-target (E:T) ratio of 1:3 in 96-well flat-bottom microplates in 200 μL RPMI media containing 10% FBS and 1% penicillin-streptomycin. Cells were incubated at 37 °C in a humidified atmosphere containing 5% CO_2, with or without caffeine (10 μM) treatment for 16 to 24 h. Culture supernatants were collected and analyzed using a human IL-2 enzyme-linked immunosorbent assay (ELISA) kit (BioLegend) according to the manufacturer's instructions. Absorbance signals were acquired using a Cytation 5 multimode plate reader (BioTek) and analyzed with Gen5 software (BioTek). IL-2 concentrations were quantified using a standard curve, and data were plotted using Prism (v10, GraphPad).

Time-lapse confocal imaging of pyroptosis using Annexin V staining

HEK293T cells were seeded on 35-mm glass-bottom dishes and transfected with GSDMDnt-B3(R44V)-P2A-COSMO-mCherry-GSDMDct (250 ng). At 16 h post-transfection, cells were stained with Pacific Blue Annexin V (1 μM) diluted in 1× binding buffer and incubated for 15 min. Immediately after caffeine addition (10 μM), time-lapse confocal imaging was initiated using the 405 nm and 561 nm channels along with bright-field imaging. Images were acquired every 15 min for 3 h. Annexin V fluorescence intensity was quantified using Nikon Elements software, and data were analyzed and plotted using Prism (v10, GraphPad).

Cytotoxicity Assays

Pyroptotic cytotoxicity was quantified using an LDH Cytotoxicity Assay Kit according to the manufacturer's instructions. HEK293T cells were seeded in 24-well plates 16 h prior to transfection and transfected with

GSDMDnt-B3(R44V)-P2A-COSMO-mCherry-GSDMDct (250 ng). For caffeine treatment group, caffeine (10 μM) was added immediately after transfection. After 24 h, 50 μL of culture supernatant was collected and transferred to a fresh 96-well flat-bottom plate.

For the maximum LDH release control, cells were lysed by adding 30 μL of lysis solution per well and incubated for 30 min, after which 50 μL of supernatant was collected. For all samples, 50 μL of working solution was added to each well, and plates were gently mixed and incubated at room temperature for 30 min in the dark. The reaction was stopped by adding 50 μL of stop solution, and absorbance at 490 nm was measured using a Cytation 5 multimode plate reader (BioTek). Data were acquired using BioTek Gen5 software and plotted using Prism (v10, GraphPad). Percent cytotoxicity was calculated as: Percent cytotoxicity = 100 x (Experimental LDH release – Baseline LDH [OD490]/maximum LDH release – Baseline LDH [OD490]).

Molecular Dynamics Simulations and Analyses

The BindCraft[6] output models were used as starting structures for our molecular dynamics simulations of the *de novo* designed binders to identify key stability indicators which would decrease the chance of false positives and increase the efficiency of our *in vitro* testing. In the case of the B3 (R44V) mutant, the Binder-COSMO complex was regenerated using AlphaFold3[7] with the mutated B3 sequence. For our molecular dynamics simulations, GROMACS 2024.4[8] was used with amber99sb-ildn force field parameters.[9] First, the models were soaked in TIP3P water with cubic box parameters and optimal salt concentrations. Then, the system was temperature-equilibrated to 300 K for 0.5 ns with a V-rescale thermostat, followed by pressure-equilibration to 1 bar for 0.5 ns with a C-rescale barostat utilizing a timestep of 2.0 femtoseconds and LINCS algorithm to constrain hydrogen bonds. After system equilibration, 4 independent 500 ns production runs of classical molecular dynamics simulations were performed for each *de novo* binder with a Nose-Hoover thermostat and a Parrinello-Rahman barostat to maintain equilibrated temperature and pressure. Following completion of the simulations, interaction energies and respective fluctuations were computed using gmx_MMPBSA[10, 11], with 1/100 frame sampling. Interface B-factors were calculated using iMOD v1.04[12] with equilibrated temperature. The final radar plot was generated in Python using NumPy[13], Pandas[14], and Matplotlib[15], with Z-scores of each descriptor normalized to [0-1].

Binder Design and *In Silico* Mutagenesis

De novo binders were designed using BindCraft[6] with target settings to generate binders of 15 – 150 amino acids, "default_4stage_multimer" advanced settings, and "default_filters" filter settings. For ΔΔG of mutation predictions, Rosetta 3.14 with ref2015 weights[16] was used to calculate changes in free energy for amino acid substitutions of each interfacing residue. Destabilizing mutations were identified by ΔΔG values of ≥ 1.0 Rosetta Energy Units (REU),

neutral mutations were identified by ΔΔG values between 1 and -1 REU, and stabilizing mutations were identified by ΔΔG values ≤ -1 REU.[17, 18] Mutations which stabilized both the *de novo* binder and binder-target complex were chosen for *in vitro* validation.

Statistics and Reproducibility

Quantitative data are reported as mean ± s.e.m., as indicated. Sample sizes (n) and the number of independent replicates for each experiment are specified in the corresponding figure legends. Statistical analyses were performed using Prism (v10, GraphPad). All cell imaging experiments were independently repeated at least three times. Statistical significance was assessed using a two-tailed Welch's t-test, with $P < 0.05$ considered significant.

Supplementary Table 1.

Supplementary Table 1| Amino acid sequences of the predicted binders and COSMO.

Binder	Sequence
**B1**	KKEIVEIIIPVMAEFMEEKKTTLSEKVFSKLETIAEAIQTYLLWFRYSNDKSIPETLEGQFEYVMEEIEKIAEEMLERI KKENDPDFEKIKEAVKEFIKYVEENVKKYL
**B2**	KQKIVEILIPIMAEFMEEKRSRLSPEVFGKLETMAEAVQTYLLWFRYSNDKSIPKTEEGIFEYVMKEIRRIIEEMLER FRKENLPELEEIERAAREFVEYVEKEAKKYL
**B3**	EPPPPTEEEIEKYELPRMQINASGAAADDVFQHTWQIFAYFYTRILERNPDLDPEELEEVMYRHFTSLIRSTDLSE EDKEWVIERFRERMRWFTEWLKR
**B4**	AALSDEEVKERLWALFEAVAKAEGTTADEEFERFQTYYMYTRDTMKTHPGILDEFRAYVQNLIDTYGEGVPAISV DEIMRFFGLT
**B5**	SFKERVEAYLDAMAEHFGVSREELYKMLEEIAKEYGWTDLEKHVLDLLHVLHAKLMEKKYGSM
**B6**	SMGEEYLEKLLELFEKFKAAKSREEKEEILREMFDLMVEVSRSRGDTDLSDEEIEQAKEIMVEAALKFFEEYPELID ELIEALKDPSKVRDFVEKHHELPTRWFNSLENPTERDKQGFHLLVQWLDFLVVVSK
**B7**	SERERVEEYLDAMAEHFGISREELYEMLEEIMKEYGWTDFEKHVLDVLHVLHAKLMERKYGSM
**B8**	SMGEEYLKKLLELLKQFKEAKSEEEKKEILREMFELMVEVSRERGDTDLSDEEIEQAKEIMVESALKFFEKYPEFL DELIEALEDPSKVREFVEKNHELPTRWFNSLKNPSEKDYQGFHLLVQWLDFLVVVSK
**B9**	MPLLEEFIEKLREIAKEAEKIPEIAELYEETARLLEEINAVEARIEPLRETDPEAAAALEREELAPLIDRLVPMFEKLK ELYFNGKKLIKTGSKYEQEFFKKHLETIYKIAMYIRMLIMSHRYPGTEEFWLAQFEYHHG
**B10**	FFAYAYQQLMEFMDEESRKLLDELWEAFVKEHKFPEEKVEAFFKATGVPEEEAKKLRELMERLAEAGKRYEEAR KSLNPDHKETPEQEEAWIAAEEAEFEFWYTLYKVVSKHMNPPP
**B11**	MLSEEEIEKIKEILKKIFPKEDEKSKKLFEVLKDFLEGKIKDVAEVFLEYAVIYGRPPTWEEFNILYKELKLPWNSVH YFMQVNFWFHDKTGQGKSIEEVKEEFLSIM
**B12**	MQRITFSKEEFERHIAIMEAGVSELEPERQAVVGAQVEALRAYYEEHGAFDMPQFHLFMHLAEEGQEIRRKAEE ERAK
**B13**	SERERIREEMREEMQRLVEKMKEVLEEVEKATGKSVEELMKEYGITFQIFLDRMRWYRWHLDREGFEKVKKELE EFLKEFKYKYTGKE
**COSMO**	MSQVQLVESGGGLVQAGGSLRLSCTASGRTGTIYSMAWFRQAPGKEREFLATVGWSSGITYYMDSVKGRFTISR DNAKNSAYLQMNSLKPEDTAVYYCTATRAWSVGYDYWGQGTQVTVS

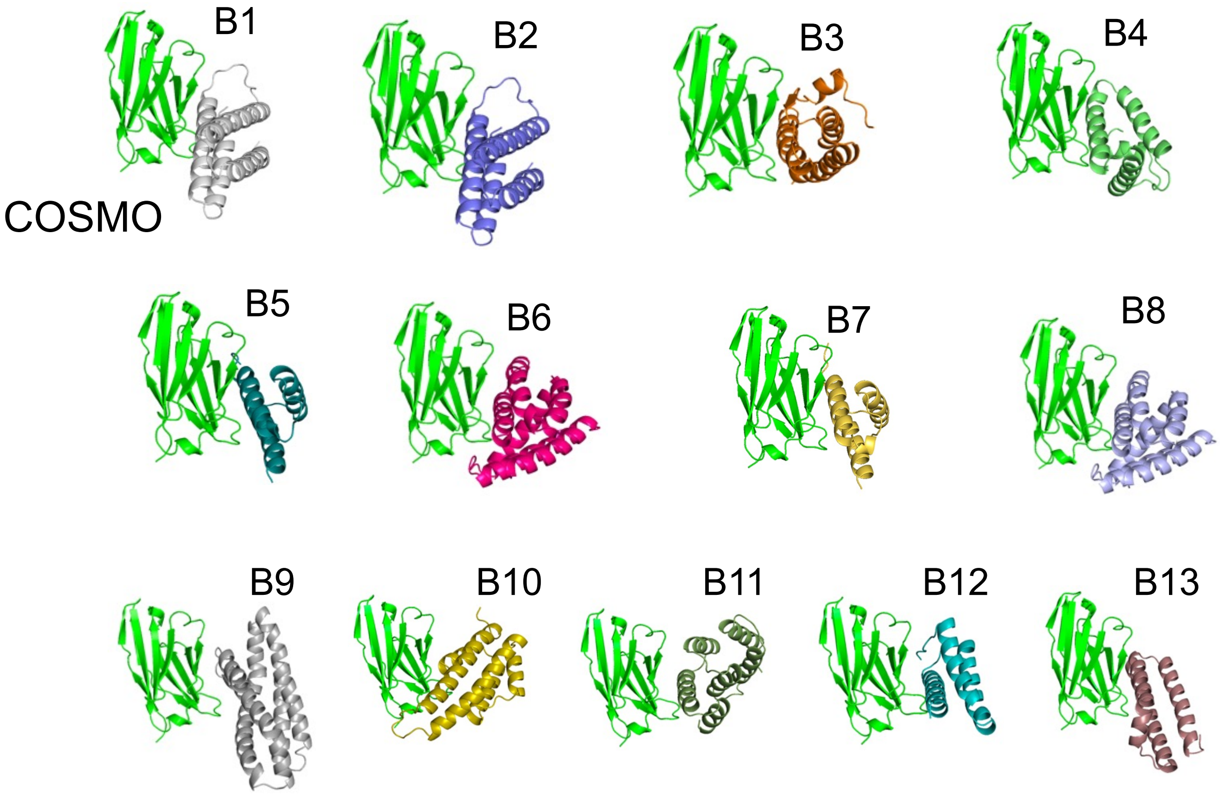

Supplementary Figure 1 | Predicted structures of COSMO-Binder complexes.
COSMO is shown in light green, and *de novo* binders are shown in various colors.

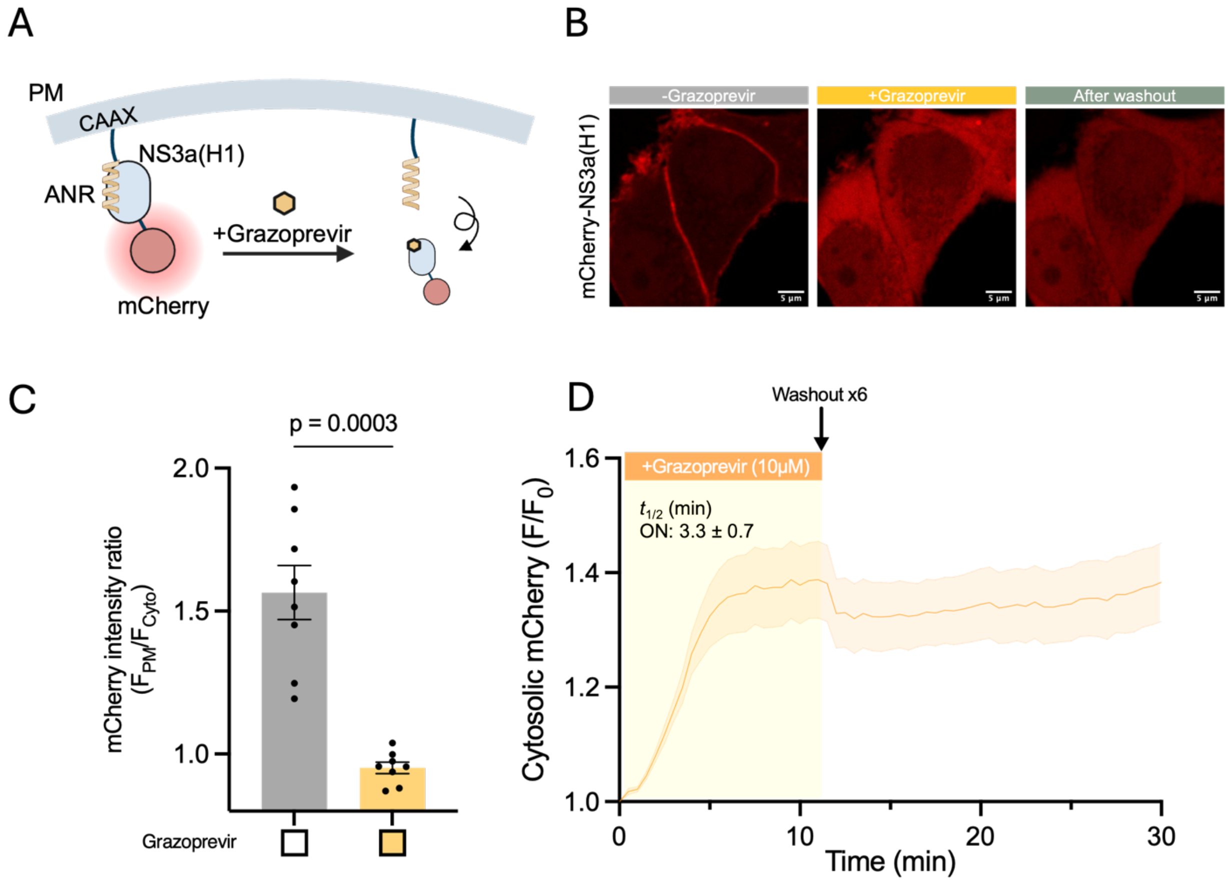

Supplementary Figure 2 | Plasma membrane-to-cytosol translocation assay for the NS3a(H1)/ANR dissociation system.

(A) Schematic illustration of the assay design. mCherry-NS3a(H1)-T2A-2×ANR-CAAX was expressed in HeLa cells to monitor ligand-induced dissociation via changes in subcellular localization. (B) Representative confocal images of HeLa cells showing the localization of mCherry-NS3a(H1) before and after addition of 10 μM Grazoprevir and following stringent washout (media replacement six times). Scale bars, 5 μm. (C) Quantification of the plasma membrane-to-cytosol fluorescence intensity ratio (F_{PM}/F_{Cyto}) of mCherry before and after Grazoprevir treatment. (D) Quantification of cytosolic mCherry fluorescence intensity in response to Grazoprevir and after washout. Data are presented as mean ± s.e.m. For quantified data (C, D), n = 8 cells per condition from three independent biological replicates. Statistical significance was determined using Welch's t-test.

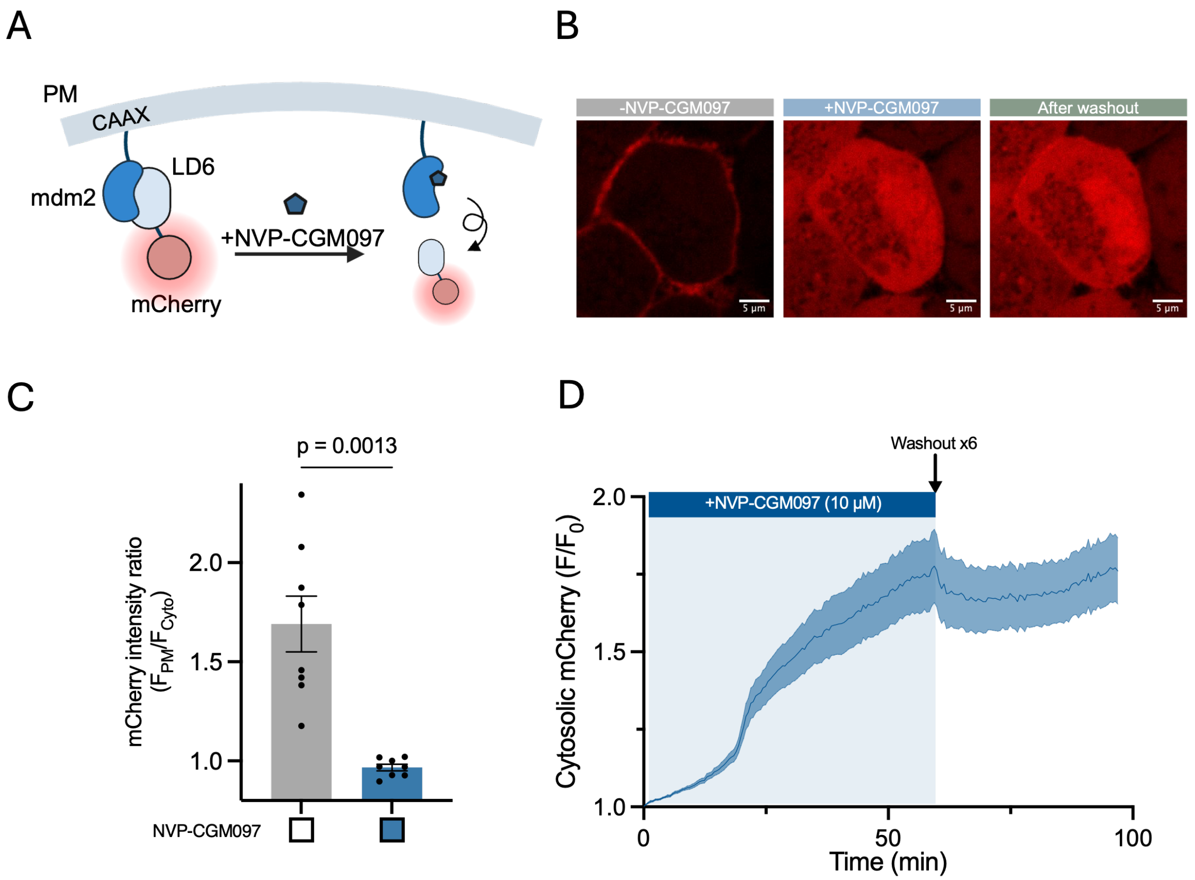

Supplementary Figure 3 | Plasma membrane-to-cytosol translocation assay for the CDH-3 dissociation system. (A) Schematic illustration of the assay design. mCherry-LD6-T2A-mdm2-CAAX was expressed in HeLa cells to monitor ligand-induced dissociation via changes in subcellular localization. (B) Representative confocal images of HeLa cells showing the localization of mCherry-LD6 before and after addition of 10 μM NVP-CGM097, and following stringent washout (media replacement six times). Scale bars, 5 μm. (C) Quantification of the plasma membrane-to-cytosol fluorescence intensity ratio (F_{PM}/F_{Cyto}) of mCherry before and after NVP-CGM097 treatment. (D) Quantification of cytosolic mCherry fluorescence intensity in response to 10 μM NVP-CGM097 and after washout. Data are presented as mean ± s.e.m. For quantified data (C, D), $n = 8$ cells per condition from three independent biological replicates. Statistical significance was determined using Welch's t-test.

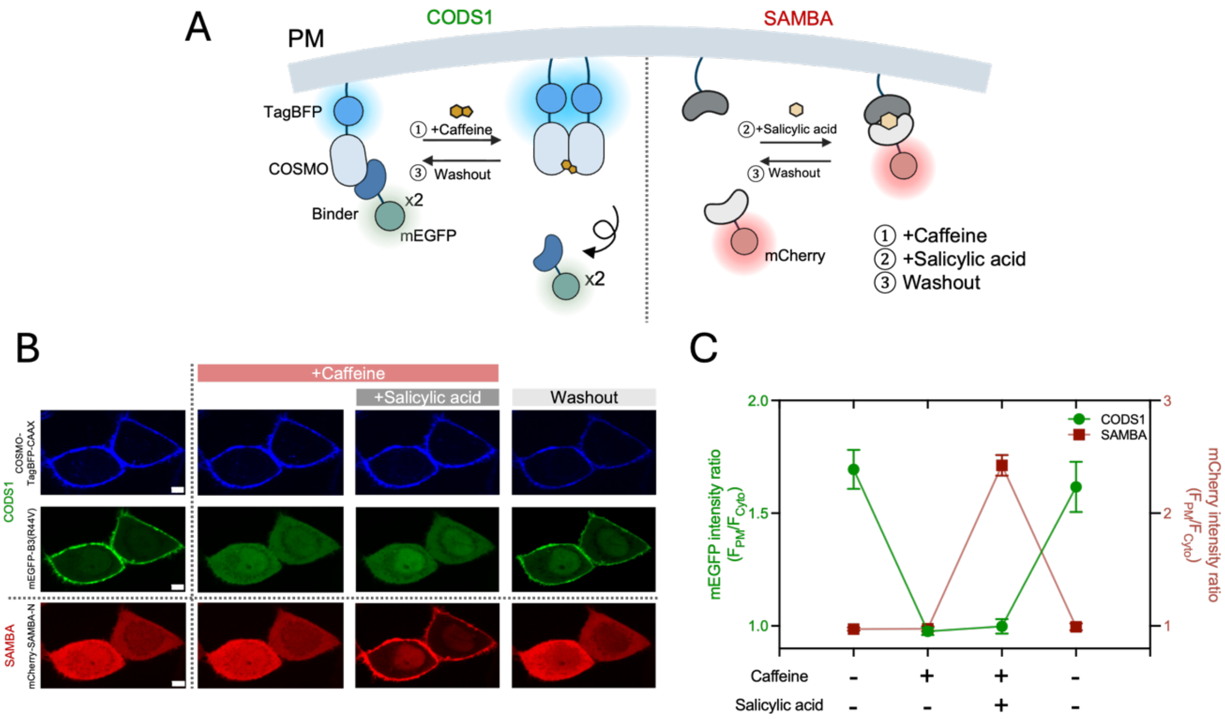

Supplementary Figure 4 | Plasma membrane-to-cytosol translocation assay evaluating the compatibility between CODS and SAMBA.

(A) Schematic illustration of the assay design. mEGFP-B3(R44V)/COSMO-TagBFP-CAAX and mCherry-SAMBA(N)-P2A-SAMBA(C)-CAAX were co-expressed in HeLa cells to assess the orthogonality between caffeine-induced dissociation (CODS) and salicylic acid-induced association (SAMBA) by monitoring the subcellular localization of the indicated components from each system. (B) Representative confocal images of HeLa cells showing the localization of mEGFP-B3(R44V) and mCherry-SAMBA(N) before and after treatment with 10 μM caffeine and 500 μM salicylic acid, as well as following washout. Scale bars, 5 μm. (C) Quantification of the plasma membrane-to-cytosol fluorescence intensity ratio (F_{PM}/F_{Cyto}) for mEGFP and mCherry signals under the indicated conditions.

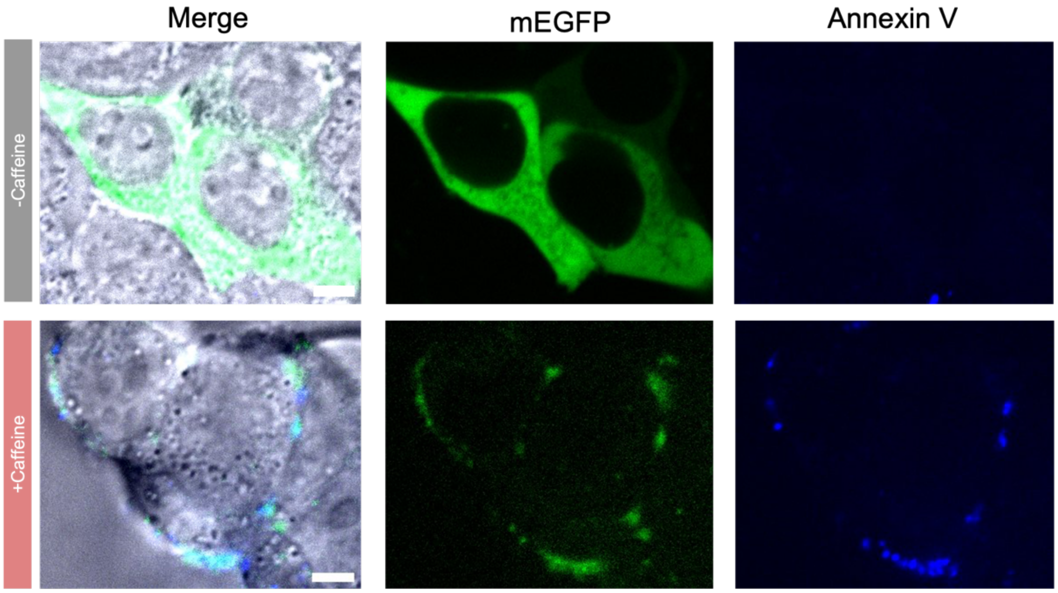

Supplementary Figure 5 | Caffeine-induced clustering of GSDMDnt-B3(R44V)-mEGFP.
Representative confocal images of HEK293T cells expressing GSDMDnt-B3(R44V)-mEGFP-P2A-COSMO-GSDMDct. Cells were treated with 10 μM caffeine, and images were acquired before and after caffeine treatment. Upon caffeine addition, CODS-mediated dissociation led to clustering of mEGFP-tagged GSDMDnt at the plasma membrane. Scale bars, 10 μm.

Supplementary Note

NLS-mCherry-TetR-biCOSMO-L

ATGGCACACCATCACCACCATCACCCCAAGAAGAAGAGGAAAGTCGGATCTGTGAGCAAGGGCGAGGAGGAT
AACATGGCCATCATCAAGGAGTTCATGCGCTTCAAGGTGCACATGGAGGGCTCCGTGAACGGCCACGAGTTC
GAGATCGAGGGCGAGGGCGAGGGCCGCCCCTACGAGGGCACCCAGACCGCCAAGCTGAAGGTGACCAAGG
GTGGCCCCCTGCCCTTCGCCTGGGACATCCTGTCCCCTCAGTTCATGTACGGCTCCAAGGCCTACGTGAAGC
ACCCCGCCGACATCCCCGACTACTTGAAGCTGTCCTTCCCCGAGGGCTTCAAGTGGGAGCGCGTGATGAACTT
CGAGGACGGCGGCGTGGTGACCGTGACCCAGGACTCCTCCCTGCAGGACGGCGAGTTCATCTACAAGGTGA
AGCTGCGCGGCACCAACTTCCCCTCCGACGGCCCCGTAATGCAGAAGAAGACCATGGGCTGGGAGGCCTCC
TCCGAGCGGATGTACCCCGAGGACGGCGCCCTGAAGGGCGAGATCAAGCAGAGGCTGAAGCTGAAGGACG
GCGGCCACTACGACGCTGAGGTCAAGACCACCTACAAGGCCAAGAAGCCCGTGCAGCTGCCCGGCGCCTAC
AACGTCAACATCAAGTTGGACATCACCTCCCACAACGAGGACTACACCATCGTGGAACAGTACGAACGCGCC
GAGGGCCGCCACTCCACCGGCGGCATGGACGAGCTGTACAAGGGAGGTAGCGGCGGTGGCGGAGGATCTG
GAGGTGGAGGTTCTTCTAGATTAGATAAAAGTAAAGTGATTAACAGCGCATTAGAGCTGCTTAATGAGGTCGGAA
TCGAAGGTTTAACAACCCGTAAACTCGCCCAGAAGCTAGGTGTAGAGCAGCCTACATTGTATTGGCATGTAAAA
AATAAGCGGGCTTTGCTCGACGCCTTAGCCATTGAGATGTTAGATAGGCACCATACTCACTTTTGCCCTTTAGAA
GGGGAAAGCTGGCAAGATTTTTTACGTAATAACGCTAAAAGTTTTAGATGTGCTTTACTAAGTCATCGCGATGGA
GCAAAAGTACATTTAGGTACACGGCCTACAGAAAAACAGTATGAAACTCTCGAAAATCAATTAGCCTTTTTATGC
CAACAAGGTTTTTCACTAGAGAATGCATTATATGCACTCAGCGCTGTGGGGCATTTTACTTTAGGTTGCGTATTG
GAAGATCAAGAGCATCAAGTCGCTAAAGAAGAAAGGGAAACACCTACTACTGATAGTATGCCGCCATTATTACG
ACAAGCTATCGAATTATTTGATCACCAAGGTGCAGAGCCAGCCTTCTTATTCGGCCTTGAATTGATCATATGCGG
ATTAGAAAAACAACTTAAATGTGAAAGTGGGTCCGGAGGTGAGAACCTGTACGGCGCGCCAAGCCAGGTGCA
ACTGGTAGAATCCGGTGGCGGTCTGGTGCAAGCGGGTGGCTCACTGCGCCTAAGCTGTACAGCATCGGGTC
GCACCGGCACCATATATTCGATGGCATGGTTTAGACAAGCGCCGGGCAAGGAACGTGAGTTTCTGGCAACTGT
CGGATGGAGCAGTGGAATAACCTACTATATGGACAGCGTGAAGGGGCGATTTACCATTAGCCGAGACAATGCA
AAGAACAGCGCTTATCTTCAGATGAACTCATTAAAGCCGGAAGATACCGCCGTGTATTATTGTACCGCAACGCG
CGCCTGGAGCGTGGGTTACGACTATTGGGGTCAGGGTACGCAGGTGACAGTATCGCATGCTGCTGCTGGCGC
TCCTGTTCCTTACCCTGATCCTCTGGAACCTCGTGAACAAAAACTGATCTCTGAAGAAGATCTGGGTGGTTCG
GGTGGCGCTCCAAGTCAAGTCCAGCTTGTTGAGTCTGGCGGCGGCCTGGTTCAAGCTGGCGGCTCTCTGCG
TCTGTCTTGCACTGCTTCTGGCCGTACTGGCACTATCTACTCTATGGCTTGGTTCCGTCAAGCTCCTGGCAAAG
AACGTGAATTCCTGGCTACTGTTGGCTGGTCTTCTGGCATCACTTACTACATGGATTCTGTTAAAGGCCGTTTCA
CTATCTCTCGTGATAACGCTAAAAACTCTGCTTACCTGCAAATGAACTCTCTGAAACCTGAAGATACTGCTGTT
TACTACTGCACTGCTACTCGTGCTTGGTCTGTTGGCTACGATTACTGGGGCCAAGGCACTCAAGTTACTGTTTC
TTAA

NLS-B3(R44V)-VP16

ATGGCACACCATCACCACCATCACCCCAAGAAGAAGAGGAAAGTCGGATCTGAACCTCCCCCTCCAACAGAA
GAAGAAATCGAGAAATATGAACTGCCTCGAATGCAAATAAACGCCTCAGGCGCAGCGGCTGATGACGTTTTCC
AGCATACGTGGCAGATATTTGCATATTTCTATACTGTTATTCTCGAAAGGAATCCGGATTTGGACCCCGAGGAGC
TTGAGGAAGTAATGTATCGCCACTTCACCAGTCTTATCCGATCAACCGACCTGTCAGAGGAAGACAAAGAGTG
GGTGATCGAGCGATTCCGAGAACGGATGAGGTGGTTCACGGAATGGCTCAAGCGAGGTGGTGGCGGCTCTG
GTGGCGGTGGCAGCGGCGGTGGTGGTTCCGGAGGCGGCGGTTCTTACTCCGGAGGTTCTGCGTACAGCCGC
GCGCGTACGAAAAACAATTACGGGTCTACCATCGAGGGCCTGCTCGATCTCCCGGACGACGACGCCCCCGAA
GAGGCGGGGCTGGCGGCTCCGCGCCTGTCCTTTCTCCCCGCGGGACACACGCGCAGACTGTCGACGGCCC
CCCCGACCGATGTCAGCCTGGGGGACGAGCTCCACTTAGACGGCGAGGACGTGGCGATGGCGCATGCCGAC
GCGCTAGACGATTTCGATCTGGACATGTTGGGGGACGGGGATTCCCCGGGTCCGGGATTTACCCCCCACGAC
TCCGCCCCCTACGGCGCTCTGGATATGGCCGACTTCGAGTTTGAGCAGATGTTTACCGATGCCCTTGGAATTG
ACGAGTACGGTGGGTAA

GSDMDnt-B3(R44V)-P2A-COSMO-mCherry-GSDMDct

ATGCCATCGGCCTTTGAGAAAGTGGTCAAGAATGTGATCAAGGAGGTAAGCGGCAGCAGAGGCGATCTCATT
CCGGTGGACAGCCTGCGGAACTCCACCAGCTTCAGGCCCTACTGCCTTCTGAACAGGAAATTTTCAAGCTCAA
GGTTCTGGAAACCCCGTTATTCATGTGTCAACCTGTCAATCAAGGACATCCTGGAGCCCAGTGCTCCAGAACC
AGAACCGGAGTGTTTTGGCTCCTTCAAAGTCTCTGATGTCGTCGATGGGAACATTCAGGGCAGAGTGATGTTG
TCAGGCATGGGAGAAGGGAAAATTTCTGGTGGGGCTGCAGTGTCTGACAGTTCCAGTGCCTCCATGAATGTG
TGTATACTGCGTGTGACTCAGAAGACCTGGGAGACCATGCAGCATGAAAGGCACCTTCAGCAGCCTGAGAAC
AAAATCCTGCAACAGCTTCGGAGTCGTGGGGATGACCTGTTTGTGGTGACCGAGGTGCTGCAGACAAAGGAG
GAAGTGCAGATCACTGAGGTCCACAGCCAAGAGGGCTCAGGCCAGTTTACGCTGCCTGGAGCTTTATGCTTG
AAGGGTGAAGGCAAGGGCCACCAAAGCCGGAAGAAGATGGTGACCATTCCTGCAGGCAGCATCCTGGCATTC
CGAGTGGCCCAACTGCTTATTGGCTCTAAATGGGATATCCTTCTCGTCTCAGATGAGAAACAGAGGACCTTTGA
GCCCTCCTCAGGTGACAGAAAAGCAGTGGGCCAGAGGCACCATGGCCTCAATGTGCTTGCTGCGCTTTGTTC
CATCGGAAAGCAGCTCAGTCTCCTGGGAGGTGGTGGCAGCGGTGGAGGAGGTGAATTCTCTGGGGGCGGTG
GCTCAGGAGAACCTCCCCCTCCAACAGAAGAAGAAATCGAGAAATATGAACTGCCTCGAATGCAAATAAACG
CCTCAGGCGCAGCGGCTGATGACGTTTTCCAGCATACGTGGCAGATATTTGCATATTTCTATACTGTTATTCTCG
AAAGGAATCCGGATTTGGACCCCGAGGAGCTTGAGGAAGTAATGTATCGCCACTTCACCAGTCTTATCCGATCA
ACCGACCTGTCAGAGGAAGACAAAGAGTGGGTGATCGAGCGATTCCGAGAACGGATGAGGTGGTTCACGGAA
TGGCTCAAGCGAAAGCTTCCACCGGTCGCCACCGGAAGCGGAGCTACTAACTTCAGCCTGCTGAAGCAGGCT
GGAGACGTGGAGGAGAACCCTGGACCTGAGCTCATGACTGAGCATGTCCGAGATGCTGCCGAGAGAGAGGG
AGTCATGCTGATTAAGAAAACTGCAGAAAATATTGATGAGGCGGCAAAAGAACTTGGTGGGGGATCCAGTCAA
GTCCAGCTTGTTGAGTCTGGCGGCGGCCTGGTTCAAGCTGGCGGCTCTCTGCGTCTGTCTTGCACTGCTTCT
GGCCGTACTGGCACTATCTACTCTATGGCTTGGTTCCGTCAAGCTCCTGGCAAAGAACGTGAATTCCTGGCTA
CTGTTGGCTGGTCTTCTGGCATCACTTACTACATGGATTCTGTTAAAGGCCGTTTCACTATCTCTCGTGATAACG
CTAAAAACTCTGCTTACCTGCAAATGAACTCTCTGAAACCTGAAGATACTGCTGTTTACTACTGCACTGCTACT
CGTGCTTGGTCTGTTGGCTACGATTACTGGGGCCAAGGCACTCAAGTTACTGTTTCTATGGTGAGCAAGGGCG
AGGAGGATAACATGGCCATCATCAAGGAGTTCATGCGCTTCAAGGTGCACATGGAGGGCTCCGTGAACGGCC
ACGAGTTCGAGATCGAGGGCGAGGGCGAGGGCCGCCCCTACGAGGGCACCCAGACCGCCAAGCTGAAGGT
GACCAAGGGTGGCCCCCTGCCCTTCGCCTGGGACATCCTGTCCCCTCAGTTCATGTACGGCTCCAAGGCCTA
CGTGAAGCACCCCGCCGACATCCCCGACTACTTGAAGCTGTCCTTCCCCGAGGGCTTCAAGTGGGAGCGCGT
GATGAACTTCGAGGACGGCGGCGTGGTGACCGTGACCCAGGACTCCTCCCTGCAGGACGGCGAGTTCATCTA
CAAGGTGAAGCTGCGCGGCACCAACTTCCCCTCCGACGGCCCCGTAATGCAGAAGAAGACCATGGGCTGGG
AGGCCTCCTCCGAGCGGATGTACCCCGAGGACGGCGCCCTGAAGGGCGAGATCAAGCAGAGGCTGAAGCTG
AAGGACGGCGGCCACTACGACGCTGAGGTCAAGACCACCTACAAGGCCAAGAAGCCCGTGCAGCTGCCCGG
CGCCTACAACGTCAACATCAAGTTGGACATCACCTCCCACAACGAGGACTACACCATCGTGGAACAGTACGAA
CGCGCCGAGGGCCGCCACTCCACCGGCGGCATGGACGAGCTGTACAAGTCAGGTTCTGGATCTGGTTCAGG
ATCACTCGAGGGATCAGGTTCTGGATCTGGTTCAGGATCAGATGGGATTGATGAGGAGGAATTAATTGAGGCG
GCAGACTTCCAGGGCCTGTATGCTGAGGTGAAGGCTTGCTCCTCAGAACTGGAGAGCTTGGAAATGGAGTTG
AGACAACAGATACTGGTGAACATCGGAAAGATTTTACAGGACCAGCCCAGCATGGAAGCCTTAGAGGCCTCAC
TAGGGCAGGGCCTGTGCAGTGGCGGCCAGGTGGAGCCTCTGGACGGCCCAGCTGGCTGCATCCTTGAGTGT
CTGGTGCTTGACTCTGGAGAACTGGTGCCGGAACTCGCAGCCCCTATCTTCTACCTGCTGGGAGCACTGGCT
GTGCTGAGTGAAACCCAGCAGCAGCTGCTAGCTAAGGCTCTGGAGACAACGGTGCTGTCAAAGCAGCTGGA
GTTGGTGAAGCACGTCTTGGAACAGAGCACCCCGTGGCAGGAGCAGAGTTCTGTGTCCCTGCCCACCGTGCT
CCTTGGGGACTGCTGGGATGAAAAGAATCCCACCTGGGTCTTGCTAGAAGAATGTGGCCTAAGGCTGCAGGT
AGAATCCCCCCAGGTGCACTGGGAACCAACGTCTCTGATCCCCACAAGTGCGCTCTATGCCTCCCTGTTCCTA
TTGTCAAGTCTAGGCCAGAAACCTTGTTAG

CODS-CAR 1B

ATGGCCTTACCAGTGACCGCCTTGCTCCTGCCGCTGGCCTTGCTGCTCCACGCCGCCAGGCCGGAACAAAAA
CTCATCTCAGAAGAGGATCTGGACATCCAGATGACACAGACTACATCCTCCCTGTCTGCCTCTCTGGGAGACA
GAGTCACCATCAGTTGCAGGGCAAGTCAGGACATTAGTAAATATTTAAATTGGTATCAGCAGAAACCAGATGGA
ACTGTTAAACTCCTGATCTACCATACATCAAGATTACACTCAGGAGTCCCATCAAGGTTCAGTGGCAGTGGGTC
TGGAACAGATTATTCTCTCACCATTAGCAACCTGGAGCAAGAAGATATTGCCACTTACTTTTGCCAACAGGGTAA
TACGCTTCCGTACACGTTCGGAGGGGGGACTAAGTTGGAAATAACAGGCTCCACCTCTGGATCCGGCAAGCC

CGGATCTGGCGAGGGATCCACCAAGGGCGAGGTGAAACTGCAGGAGTCAGGACCTGGCCTGGTGGCGCCC
TCACAGAGCCTGTCCGTCACATGCACTGTCTCAGGGGTCTCATTACCCGACTATGGTGTAAGCTGGATTCGCC
AGCCTCCACGAAAGGGTCTGGAGTGGCTGGGAGTAATATGGGGTAGTGAAACCACATACTATAATTCAGCTCT
CAAATCCAGACTGACCATCATCAAGGACAACTCCAAGAGCCAAGTTTTCTTAAAAATGAACAGTCTGCAAACTG
ATGACACAGCCATTTACTACTGTGCCAAACATTATTACTACGGTGGTAGCTATGCTATGGACTACTGGGGTCAA
GGAACCTCAGTCACCGTCTCCTCAGCGGCCGCAACCACGACGCCAGCGCCGCGACCACCAACACCGGCGCC
CACCATCGCGTCACAGCCCCTGTCCCTGCGCCCAGAGGCGTGCCGGCCAGCGGCGGGGGGCGCAGTGCAC
ACGAGGGGGCTGGACTTCGCCTGTGATATCTACATCTGGGCGCCCTTGGCCGGGACTTGTGGGGTCCTTCTC
CTGTCACTGGTTATCACCCTTTACTGCAAACGGGGCAGAAAGAAACTCCTGTATATATTCAAACAACCATTTATG
AGACCAGTACAAACTACTCAAGAGGAAGATGGCTGTAGCTGCCGATTTCCAGAAGAAGAAGAAGGAGGATGT
GAAGGTAGTGGTAGTGGATCTTCCCTATCCCGGGATCCACCGGTCGCCACCAAGAGCAGGATCACCAGCGAG
GGCGAGTACATCCCCCTGGACCAGATCGACATCAACGTGACGCGTATGGTGAGCAAGGGCGAGGAGCTGTTC
ACCGGGGTGGTGCCCATCCTGGTCGAGCTGGACGGCGACGTAAACGGCCACAAGTTCAGCGTGTCCGGCGA
GGGCGAGGGCGATGCCACCTACGGCAAGCTGACCCTGAAGTTCATCTGCACCACCGGCAAGCTGCCCGTGC
CCTGGCCCACCCTCGTGACCACCTTCGGCTACGGCCTGCAGTGCTTCGCCCGCTACCCCGACCACATGAAGC
AGCACGACTTCTTCAAGTCCGCCATGCCCGAAGGCTACGTCCAGGAGCGCACCATCTTCTTCAAGGACGACGG
CAACTACAAGACCCGCGCCGAGGTGAAGTTCGAGGGCGACACCCTGGTGAACCGCATCGAGCTGAAGGGCA
TCGACTTCAAGGAGGACGGCAACATCCTGGGGCACAAGCTGGAGTACAACTACAACAGCCACAACGTCTATA
TCATGGCCGACAAGCAGAAGAACGGCATCAAGGTGAACTTCAAGATCCGCCACAACATCGAGGACGGCAGCG
TGCAGCTCGCCGACCACTACCAGCAGAACACCCCCATCGGCGACGGCCCCGTGCTGCTGCCCGACAACCAC
TACCTGAGCTACCAGTCCGCCCTGAGCAAAGACCCCAACGAGAAGCGCGATCACATGGTCCTGCTGGAGTTC
GTGACCGCCGCCGGGATCACTCTCGGCATGGACGAGCTGTACAAGGATATCTTCTGCTACGAGAACGAGGTG
GCGGCCGCGACTCTAGATCATAATCAGGGAAGCGGGTCCGGTAGCGGCGCGCCAGAACCTCCCCCTCCAAC
AGAAGAAGAAATCGAGAAATATGAACTGCCTCGAATGCAAATAAACGCCTCAGGCGCAGCGGCTGATGACGT
TTTCCAGCATACGTGGCAGATATTTGCATATTTCTATACTGTTATTCTCGAAAGGAATCCGGATTTGGACCCCGA
GGAGCTTGAGGAAGTAATGTATCGCCACTTCACCAGTCTTATCCGATCAACCGACCTGTCAGAGGAAGACAAA
GAGTGGGTGATCGAGCGATTCCGAGAACGGATGAGGTGGTTCACGGAATGGCTCAAGCGATAG

CODS-CAR 2B

ATGAATGAACTGGCACTGAAGCTCGCGGGTCTTGACCTGGGAGGCTCCGATCCGCCGGTCGCCACCCTCGA
GATGATCCATCTGGGTCACATCCTCTTCCTGCTTTTGCTCCCAGTGGCTGCAGCTCAGACGACTCCAGGAGAG
AGATCATCACTCCCTGCCTTTTACCCTGGCACTTCAGGCTCTTGTTCCGGATGTGGGTCCCTCTCTCTGCCGAT
CTACATCTGGGCGCCCTTGGCCGGGACTTGTGGGGTCCTTCTCCTGTCACTGGTTATCACCCTTTACTGCTCT
CTGGGTACCAAACGGGGCAGAAAGAAACTCCTGTATATATTCAAACAACCATTTATGAGACCAGTACAAACTAC
TCAAGAGGAAGATGGCTGTAGCTGCCGATTTCCAGAAGAAGAAGAAGGAGGATGTGAACTGAGAGTGAAGTT
CAGCAGGAGCGCAGACGCCCCCGCGTACAAGCAGGGCCAGAACCAGCTCTATAACGAGCTCAATCTAGGAC
GAAGAGAGGAGTACGATGTTTTGGACAAGAGACGTGGCCGGGACCCTGAGATGGGGGGAAAGCCGAGAAGG
AAGAACCCTCAGGAAGGCCTGTACAATGAACTGCAGAAAGATAAGATGGCGGAGGCCTACAGTGAGATTGGG
ATGAAAGGCGAGCGCCGGAGGGGCAAGGGGCACGATGGCCTTTACCAGGGTCTCAGTACAGCCACCAAGGA
CACCTACGACGCCCTTCACATGCAGGCCCTGCCCCCTCGCTCGCGAGGAAGCGGGTCCGGTAGCGGATCTG
AATTCATGGTGAGCAAGGGCGAGGAGGATAACATGGCCATCATCAAGGAGTTCATGCGCTTCAAGGTGCACAT
GGAGGGCTCCGTGAACGGCCACGAGTTCGAGATCGAGGGCGAGGGCGAGGGCCGCCCCTACGAGGGCACC
CAGACCGCCAAGCTGAAGGTGACCAAGGGTGGCCCCCTGCCCTTCGCCTGGGACATCCTGTCCCCTCAGTTC
ATGTACGGCTCCAAGGCCTACGTGAAGCACCCCGCCGACATCCCCGACTACTTGAAGCTGTCCTTCCCCGAG
GGCTTCAAGTGGGAGCGCGTGATGAACTTCGAGGACGGCGGCGTGGTGACCGTGACCCAGGACTCCTCCCT
GCAGGACGGCGAGTTCATCTACAAGGTGAAGCTGCGCGGCACCAACTTCCCCTCCGACGGCCCCGTAATGCA
GAAGAAGACCATGGGCTGGGAGGCCTCCTCCGAGCGGATGTACCCCGAGGACGGCGCCCTGAAGGGCGAG
ATCAAGCAGAGGCTGAAGCTGAAGGACGGCGGCCACTACGACGCTGAGGTCAAGACCACCTACAAGGCCAA
GAAGCCCGTGCAGCTGCCCGGCGCCTACAACGTCAACATCAAGTTGGACATCACCTCCCACAACGAGGACTA
CACCATCGTGGAACAGTACGAACGCGCCGAGGGCCGCCACTCCACCGGCGGCATGGACGAGCTGTACAAGT
ATGGTGGATCAGGCGCGCCAAGTCAAGTCCAGCTTGTTGAGTCTGGCGGCGGCCTGGTTCAAGCTGGCGGCT
CTCTGCGTCTGTCTTGCACTGCTTCTGGCCGTACTGGCACTATCTACTCTATGGCTTGGTTCCGTCAAGCTCCT
GGCAAAGAACGTGAATTCCTGGCTACTGTTGGCTGGTCTTCTGGCATCACTTACTACATGGATTCTGTTAAAGG

CCGTTTCACTATCTCTCGTGATAACGCTAAAAACTCTGCTTACCTGCAAATGAACTCTCTGAAACCTGAAGATA
CTGCTGTTTACTACTGCACTGCTACTCGTGCTTGGTCTGTTGGCTACGATTACTGGGGCCAAGGCACTCAAGTT
ACTGTTTCTTAG

Caption for Supplementary Video

Supplementary Video 1 | Caffeine-induced dissociation of B3(R44V) from plasma membrane-anchored biCOSMO-L. Time-lapse imaging of HeLa cells co-expressing biCOSMO-L-mEGFP–CAAX and B3(R44V)-mCherry following treatment with 10 μM caffeine.

References

(1) Wang, T.; He, L.; Jing, J.; Lan, T. H.; Hong, T.; Wang, F.; Huang, Y.; Ma, G.; Zhou, Y. Caffeine-Operated Synthetic Modules for Chemogenetic Control of Protein Activities by Life Style. *Adv Sci (Weinh)* **2021**, *8* (3), 2002148. DOI: 10.1002/advs.202002148.

(2) He, L.; Huang, Z.; Huang, K.; Chen, R.; Nguyen, N. T.; Wang, R.; Cai, X.; Huang, Z.; Siwko, S.; Walker, J. R.; et al. Optogenetic Control of Non-Apoptotic Cell Death. *Adv Sci (Weinh)* **2021**, *8* (13), 2100424. DOI: 10.1002/advs.202100424.

(3) Wang, T.; Liu, S.; Ke, Y.; Ali, S.; Wang, R.; Hong, T.; Liu, Z.; Ma, G.; Lan, T. H.; Wang, F.; et al. Repurposing salicylic acid as a versatile inducer of proximity. *Nat Chem Biol* **2025**, *21* (9), 1444-1456. DOI: 10.1038/s41589-025-01918-z.

(4) Stringer, C.; Wang, T.; Michaelos, M.; Pachitariu, M. Cellpose: a generalist algorithm for cellular segmentation. *Nat Methods* **2021**, *18* (1), 100-106. DOI: 10.1038/s41592-020-01018-x.

(5) Stringer, C.; Pachitariu, M. Cellpose3: one-click image restoration for improved cellular segmentation. *Nat Methods* **2025**, *22* (3), 592-599. DOI: 10.1038/s41592-025-02595-5.

(6) Pacesa, M.; Nickel, L.; Schellhaas, C.; Schmidt, J.; Pyatova, E.; Kissling, L.; Barendse, P.; Choudhury, J.; Kapoor, S.; Alcaraz-Serna, A.; et al. One-shot design of functional protein binders with BindCraft. *Nature* **2025**, *646* (8084), 483-492. DOI: 10.1038/s41586-025-09429-6.

(7) Abramson, J.; Adler, J.; Dunger, J.; Evans, R.; Green, T.; Pritzel, A.; Ronneberger, O.; Willmore, L.; Ballard, A. J.; Bambrick, J.; et al. Accurate structure prediction of biomolecular interactions with AlphaFold 3. *Nature* **2024**, *630* (8016), 493-500. DOI: 10.1038/s41586-024-07487-w.

(8) Pronk, S.; Páll, S.; Schulz, R.; Larsson, P.; Bjelkmar, P.; Apostolov, R.; Shirts, M. R.; Smith, J. C.; Kasson, P. M.; van der Spoel, D.; et al. GROMACS 4.5: a high-throughput and highly parallel open source molecular simulation toolkit. *Bioinformatics* **2013**, *29* (7), 845-854. DOI: 10.1093/bioinformatics/btt055.

(9) Lindorff-Larsen, K.; Piana, S.; Palmo, K.; Maragakis, P.; Klepeis, J. L.; Dror, R. O.; Shaw, D. E. Improved side-chain torsion potentials for the Amber ff99SB protein force field. *Proteins* **2010**, *78* (8), 1950-1958. DOI: 10.1002/prot.22711.

(10) Valdés-Tresanco, M. S.; Valdés-Tresanco, M. E.; Valiente, P. A.; Moreno, E. gmx_MMPBSA: A New Tool to Perform End-State Free Energy Calculations with GROMACS. *J Chem Theory Comput* **2021**, *17* (10), 6281-6291. DOI: 10.1021/acs.jctc.1c00645.

(11) Miller, B. R., 3rd; McGee, T. D., Jr.; Swails, J. M.; Homeyer, N.; Gohlke, H.; Roitberg, A. E. MMPBSA.py: An Efficient Program for End-State Free Energy Calculations. *J Chem Theory Comput* **2012**, *8* (9), 3314-3321. DOI: 10.1021/ct300418h.

(12) Lopéz-Blanco, J. R.; Garzón, J. I.; Chacón, P. iMod: multipurpose normal mode analysis in internal coordinates. *Bioinformatics* **2011**, *27* (20), 2843-2850. DOI: 10.1093/bioinformatics/btr497.
(13) Harris, C. R.; Millman, K. J.; van der Walt, S. J.; Gommers, R.; Virtanen, P.; Cournapeau, D.; Wieser, E.; Taylor, J.; Berg, S.; Smith, N. J.; et al. Array programming with NumPy. *Nature* **2020**, *585* (7825), 357-362. DOI: 10.1038/s41586-020-2649-2.
(14) McKinney, W. Data Structures for Statistical Computing in Python. *SciPy* **2010**. DOI: https://doi.org/10.25080/Majora-92bf1922-00a.
(15) Cabral, M. L. Matplotlib: A 2D Graphics Environment. *Computing in Science & Engineering* **2007**, *9* (3), 90-95. DOI: 10.1109/MCSE.2007.55.
(16) Alford, R. F.; Leaver-Fay, A.; Jeliazkov, J. R.; O'Meara, M. J.; DiMaio, F. P.; Park, H.; Shapovalov, M. V.; Renfrew, P. D.; Mulligan, V. K.; Kappel, K.; et al. The Rosetta All-Atom Energy Function for Macromolecular Modeling and Design. *J Chem Theory Comput* **2017**, *13* (6), 3031-3048. DOI: 10.1021/acs.jctc.7b00125.
(17) Mavor, D.; Barlow, K.; Thompson, S.; Barad, B. A.; Bonny, A. R.; Cario, C. L.; Gaskins, G.; Liu, Z.; Deming, L.; Axen, S. D.; et al. Determination of ubiquitin fitness landscapes under different chemical stresses in a classroom setting. *Elife* **2016**, *5*. DOI: 10.7554/eLife.15802.
(18) Sapozhnikov, Y.; Patel, J. S.; Ytreberg, F. M.; Miller, C. R. Statistical modeling to quantify the uncertainty of FoldX-predicted protein folding and binding stability. *BMC Bioinformatics* **2023**, *24* (1), 426. DOI: 10.1186/s12859-023-05537-0.
